# Immune escape of colorectal tumours via local LRH‐1/Cyp11b1‐mediated synthesis of immunosuppressive glucocorticoids

**DOI:** 10.1002/1878-0261.13414

**Published:** 2023-03-09

**Authors:** Asma Ahmed, Cindy Reinhold, Eileen Breunig, Truong San Phan, Lea Dietrich, Feodora Kostadinova, Corinne Urwyler, Verena M. Merk, Mario Noti, Israel Toja da Silva, Konstantin Bode, Fatima Nahle, Anna Pia Plazzo, Julia Koerner, Regula Stuber, Constantin Menche, Eva Karamitopoulou, Henner F. Farin, Kenneth J. Gollob, Thomas Brunner

**Affiliations:** ^1^ Division of Biochemical Pharmacology, Department of Biology University of Konstanz Germany; ^2^ Department of Pharmacology, Faculty of Medicine University of Khartoum Sudan; ^3^ Department of Biology, Institute of Molecular Health Sciences Swiss Federal Institute of Technology (ETH) Zurich Switzerland; ^4^ Institute of Pathology University of Bern Switzerland; ^5^ International Research Center, A.C. Camargo Cancer Center São Paulo Brazil; ^6^ National Institute for Science and Technology – Oncogenomics and Therapeutic Innovation (INCT‐INOTE) São Paulo Brazil; ^7^ Division of Immunology, Department of Biology University of Konstanz Germany; ^8^ Georg‐Speyer‐Haus, Institute for Tumor Biology and Experimental Therapy Frankfurt am Main Germany; ^9^ Frankfurt Cancer Institute Goethe University Frankfurt am Main Germany; ^10^ Albert Einstein Israelite Hospital São Paulo Brazil; ^11^ Present address: Cancer Research UK Beatson Institute Bearsden G611BD UK; ^12^ Present address: LHO Laboratory for Hematology and Oncology 68161 Mannheim Germany; ^13^ Present address: Société des Produits Nestlé, Nestlé Research 1000 Lausanne Switzerland

**Keywords:** colorectal cancer, CYP11B1, glucocorticoids, immune checkpoint, liver receptor homolog‐1 (NR5A2), tumour immune escape

## Abstract

Control of tumour development and growth by the immune system critically defines patient fate and survival. What regulates the escape of colorectal tumours from destruction by the immune system remains currently unclear. Here, we investigated the role of intestinal synthesis of glucocorticoids in the tumour development during an inflammation‐induced mouse model of colorectal cancer. We demonstrate that the local synthesis of immunoregulatory glucocorticoids has dual roles in the regulation of intestinal inflammation and tumour development. In the inflammation phase, LRH‐1/Nr5A2‐regulated and Cyp11b1‐mediated intestinal glucocorticoid synthesis prevents tumour development and growth. In established tumours, however, tumour‐autonomous Cyp11b1‐mediated glucocorticoid synthesis suppresses anti‐tumour immune responses and promotes immune escape. Transplantation of glucocorticoid synthesis‐proficient colorectal tumour organoids into immunocompetent recipient mice resulted in rapid tumour growth, whereas transplantation of Cyp11b1‐deleted and glucocorticoid synthesis‐deficient tumour organoids was characterized by reduced tumour growth and increased immune cell infiltration. In human colorectal tumours, high expression of steroidogenic enzymes correlated with the expression of other immune checkpoints and suppressive cytokines, and negatively correlated with overall patients' survival. Thus, LRH‐1‐regulated tumour‐specific glucocorticoid synthesis contributes to tumour immune escape and represents a novel potential therapeutic target.

## Graphical abstract

Extra‐adrenal glucocorticoid synthesis in the intestinal epithelium controls intestinal homeostasis, inflammation and colitis‐associated tumour development in a Liver Receptor Homolog‐1 (LRH‐1/NR5A2)‐regulated manner. LRH‐1 drives proliferation and tumour‐autonomous glucocorticoid synthesis, which suppresses anti‐tumour immune responses and promotes immune escape.

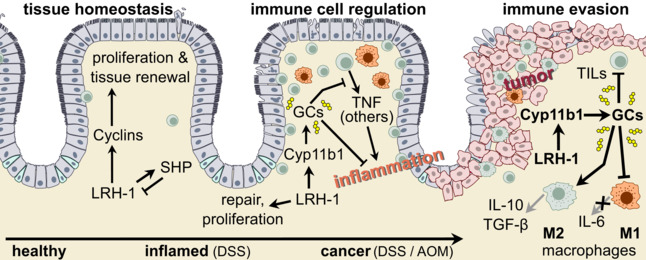

**Abbreviations**
AOM: azoxymethane
Ccnd1: cyclin D1
Ccne1: cyclin E1
CTLA‐4: cytotoxic T lymphocyte‐associated antigen 4
CYP: cytochrome p450
CYP11B1: 11β‐hydroxylase
DSS: dextran sodium sulphate
IL: interleukin
LRH‐1: liver receptor homolog‐1 (NR5A2)
MHC: major histocompatibility complex
NK: natural killer
PD1: programmed death 1
SF‐1: steroidogenic factor‐1 (NR5A1)
SHP: small heterodimer partner (NR0B2)
TGFβ: transforming growth factor β
TNF: tumour necrosis factor
T_reg_
: regulatory T cells

## 1 Introduction

The control of tumour development and growth by the immune system has been first suggested more than 50 years ago [1]; however, its physiological relevance has been questioned for decades. Though many tumours express tumour‐specific or tumour‐associated antigens, and tumour patients have tumour‐specific T cells, it has been unclear if and how much these immune effector cells contribute to the control of tumour growth. More recent studies, however, strongly support the idea that immune surveillance critically limits tumour development and growth. In colorectal tumour patients, infiltration of tumours with effector memory CD8^+^ T cells has been associated with increased patient survival [2, 3]. This has led to the development of a so‐called “immunoscore”, which stratifies colorectal tumour patients based on the extent and quality of immune cell infiltrates (reviewed in Ref. [4]). The tumour microenvironment is, however, not only populated by tumour‐controlling cytotoxic T cells, but also by other types of immune cells, which may even support tumour development by either suppressing the immune response or by promoting tumour vascularization and thereby tumour growth. Colorectal tumours are often associated with increased numbers of FoxP3^+^ regulatory T cells (T_reg_), which may limit protective immune surveillance [5]. Similarly, tumour‐associated macrophages are frequently present in the microenvironment of colorectal tumours. While pro‐inflammatory M1‐like macrophages are correlated with increased survival, the presence of anti‐inflammatory M2‐like macrophages is associated with poor prognosis (reviewed in Ref. [6]).

These findings suggest that the ability of tumours to condition their microenvironment may largely determine whether or not the immune system is able to control tumour growth and metastases. Thus, tumour‐derived factors, which limit the infiltration, activation or even survival of tumour‐specific T lymphocytes, likely substantially support tumour growth and immune evasion. The “tool kit” of cancer cells in this regard is rather extensive. Tumour cells downregulate major histocompatibility complex (MHC) class I to prevent recognition by cytotoxic T cells [7], release transforming growth factor β (TGFβ) to suppress their activation [8] or express cytotoxic effector molecules usually restricted to cytotoxic lymphocytes, e.g., Fas (CD95) ligand, and thereby induce apoptosis in tumour‐infiltrating immune cells [9, 10]. Tumour cells also hijack regulatory mechanisms, which usually contribute to the regulation of immune tolerance, and thereby prevent excessive tissue damage. The inhibitory receptors cytotoxic T‐lymphocyte‐associated antigen 4 (CTLA‐4) and programmed death 1 (PD1) generally interact with their cognate ligands CD80 and PD1L, respectively, on antigen‐presenting cells. Balanced activation of these inhibitory receptors critically contributes to the maintenance of immune homeostasis, as evidenced by the development of autoimmune disease in CTLA‐4‐ and PD1‐deficient mice. The same inhibitory receptors, however, also prevent the activation of tumour‐specific T cells. This is why anti‐PD1L and anti‐CTLA‐4 antibodies have a therapeutic effect in tumour patients, in particular when applied in combination with cell death‐promoting agents, likely by enhancing immunological cell death [11].

Yet, not all patients respond to immune checkpoint inhibitors, i.e., anti‐PD‐L1 and anti‐CTLA‐4 antibodies. This suggests that different immunoregulatory mechanisms exist, which prevent the destruction of tumours by the immune system. Glucocorticoids are steroid hormones with potent immunosuppressive activities. Since their discoveries in the 1950s, they could not be missed in the treatment of numerous devastating inflammatory and autoimmune diseases. Endogenous glucocorticoids are primarily produced in the adrenal glands in response to emotional, physical and inflammatory stress. In the last two decades, various alternative sources of glucocorticoids, such as thymus, skin, lung, vasculature and intestine, have been discovered (reviewed in Ref. [12, 13, 14]). In the intestine, we identified intestinal crypts as the relevant source of glucocorticoids [15]. Interestingly, the regulation of intestinal and adrenal glucocorticoid synthesis differs substantially. While in the adrenal glands, steroidogenesis is under the transcriptional control of the nuclear receptor, Steroidogenic Factor‐1 (NR5A1), in the intestine, it is functionally replaced by its close homolog LRH‐1 (Liver Receptor Homolog‐1, NR5A2) [16, 17, 18]. Intestine‐specific deletion of LRH‐1 abrogates immunological stress‐driven intestinal glucocorticoid synthesis and sensitizes for experimentally induced colitis [18, 19]. Similarly, the deletion of LRH‐1 results in exacerbated immune responses against intestinal viral infections [15, 20]. Of interest, LRH‐1 not only regulates intestinal glucocorticoid synthesis but is also critically involved in intestinal epithelial homeostasis and renewal [21]. LRH‐1 is expressed predominantly in the intestinal crypts where it regulates the transcription of cyclin E1 and D1, and thereby the proliferation of stem and early progenitor cells [22]. Given its importance in the regulation of cell cycle, LRH‐1 has been proposed as an oncogene. Indeed, LRH‐1 is frequently overexpressed in colorectal tumours [23, 24], and genetic deletion of LRH‐1 results in reduced development of tumours in murine models of colorectal cancer [25].

Recently, we have shown that human colorectal tumours have maintained the capability to produce immunoregulatory glucocorticoids in an LRH‐1‐dependent manner [24]. This suggests that LRH‐1 may have a dual role in the development of colorectal cancer. On one hand, it may support tumour proliferation via the transcriptional control of cell cycle‐regulating genes, on the other hand, it may promote immune evasion by suppressing anti‐tumour immune responses via the synthesis of immunoregulatory glucocorticoids [26]. However, experimental evidence for the latter hypothesis has been lacking to date. Here, we demonstrate that LRH‐1 and its inhibitor SHP (Small Heterodimer Partner, NR0B2) are critically involved in the development of inflammation‐driven intestinal tumours, by regulating the initial inflammatory phase, which promotes tumour development, as well as the immune escape of established tumours. Employing mice incapable of producing intestinal glucocorticoids, and 3D tumour organoid transplantation, we demonstrate that suppressing anti‐tumour immune responses by tumour‐derived glucocorticoids is critical for successful tumour growth and development. Finally, gene expression analysis in tumours from human colorectal patients reveals that the expression of genes controlling glucocorticoid synthesis significantly correlates with the expression of immunoregulatory gene products and overall patient survival. Thus, tumour‐derived glucocorticoids may represent a novel immune checkpoint and attractive therapeutic target in the treatment of colorectal tumours.

## 2 Materials and methods

### 2.1 Cell lines

The cell line HEK293T had been originally obtained from Inder Verma (Salk Institute, Ja Jolla, CA, USA). The authentication of the cell line was tested by PCR confirming the presence of the SV40 large T antigen. The murine intestinal epithelial cell line mICcl2 was obtained from J.‐P. Kraehenbuehl (Lausanne). The authentication of the cell line was confirmed by flow cytometry confirming E‐cadherin expression. All experiments were conducted with mycoplasma‐free cell lines.

### 2.2 Animal studies

All animal experiments were approved by the local ethics committee (Regierungspräsidium Freiburg, animal license number G15‐88, G17‐163) and conducted according to ARRIVE standards. Age‐ and sex‐matched 7‐ to 11‐week‐old mice were used for the experiments. Mice were housed in the animal facility of the University of Konstanz under specific pathogen‐free conditions with a standard diet and water *ad libitum*. All mice were bred under the C57BL/6 genetic background. SHP‐deficient mice (SHP^−/−^) [27] and RAG1‐deficient mice (*Rag1* ^−/−^) [28] had been described previously. Intestinal epithelial cell‐specific LRH‐1 knockout mice (villin‐Cre x LRH‐1^fl/fl^, LRH‐1^IEC KO^) were generated by crossing LRH‐1^fl/fl^ mice with villin‐Cre transgenic mice [16]. The littermates lacking the Cre expression (LRH‐1^fl/fl^) were used as controls. LRH‐1^IEC KO^ and SHP‐deficient mice were kindly provided by K. Schoonjans (EPFL Lausanne, Switzerland). For all experiments, sibling littermates were used as controls, with the exception of SHP^−/−^ mice, for which wild‐type C57Bl/6 mice, housed in the same room, were used.

### 2.3 Generation of intestine‐specific Cyp11b1 knockout mice

The floxed Cyp11b1 mouse line was generated in the Institute Clinique de la Souris in Illkirch, France by targeting exons 3–5 of the *Cyp11b1* gene with loxP sites [29]. The resulting Cyp11b1^fl/fl^ mouse line was crossed with villin‐Cre transgenic mice to generate the intestine‐specific Cyp11b1‐deficient mouse line (Cyp11b1^IEC KO^). The expression of Cre and the successful deletion of floxed exons 3–5 in the small and large intestines was confirmed by PCR (Fig. S1) using the primers listed in Table S1.

### 2.4 Differential isolation of intestinal epithelial cells along the villus–crypt axis

Intestinal epithelial cells from the small intestine of wild‐type mice were differentially isolated in seven fractions from villus to crypts as described previously [15], and used for quantification of LRH‐1 target genes by quantitative RT‐PCR. For the comparison between wild‐type and *Shp* ^−/−^ mice, four fractions from villus to crypts were isolated.

### 2.5 AOM/DSS inflammation‐driven colon carcinogenesis model

The AOM/DSS colon carcinogenesis model was performed as described previously [30]. Briefly, female mice were injected i.p. with 12 mg·kg^−1^ body weight azoxymethane (AOM) (Sigma‐Aldrich, St. Louis, MO, USA). Five days later, 2.2% (w/v) dextran sulphate sodium (DSS) (molecular weight 36–50 kDa; MP Biomedicals, Eschwege, Germany) was given via the drinking water for five consecutive days, followed by 16 days of normal drinking water. This DSS cycle was repeated 2, resp. 3 times. Body weights were monitored. At time points, indicated mice were sacrificed, and colons were removed for macroscopic examination of tumour numbers and size, measurement of colon length, total RNA isolation and histological or immunohistochemical analysis. Tumour diameter was measured with a sliding calliper and the volume was calculated using the following formula: *V* = 4/3π*r* ^3^ (*V*: volume, π constant = 3.14 and *r*: radius = diameter/2).

### 2.6 Induction of acute colitis

Female wild‐type and SHP^−/−^ mice were treated with 2.2% (w/v) DSS in the drinking water for 5 days followed by normal drinking water. Control mice received normal drinking water. Body weights were recorded. Mice were sacrificed on days 5, 7 and 10 after DSS treatment. Colons were longitudinally divided for RNA extraction, histological analysis (“Swiss rolls”) and *ex vivo* culture for measurement of colonic glucocorticoid synthesis [16].

### 2.7 Histopathological and immunohistochemical analyses of colonic tissues

Colonic mouse tissues were fixed in 10% formalin and embedded in paraffin. Paraffin‐embedded “Swiss roll” sections were stained with H&E for histopathological evaluation and scored microscopically for colitis using a scoring method described previously [31], ranging from 0 to a maximal score of 40.

### 2.8 Detection of colonic glucocorticoid synthesis

Analysis of colonic glucocorticoid synthesis was done as described previously [15, 18, 32] using a radioimmunoassay. Glucocorticoid synthesis was normalized to tissue weight.

### 2.9 Colorectal tumour organoids culture

Tumour organoids from wild‐type, SHP^−/−^, LRH‐1^fl/fl^, LRH‐1^IEC KO^, Cyp11b1^fl/fl^ and Cyp11b1^IEC KO^ mice were generated as described previously [33, 34] with minor modification. In brief, tumours were isolated from AOM/DSS‐treated mice at day 56 and digested in digestion buffer. Single cells were washed, seeded into 24‐well plates at a density of 15 000 cells per 50 μL Basement Membrane Matrix (BME; Trevigen, Gaithersburg, MD, USA) per well and overlaid with 500 μL tumour organoid medium [35]. The culture medium was changed every 2 days. Glucocorticoid synthesis was analysed as described above.

For the analysis of tumour organoid growth, equal numbers of cells were seeded into a 96‐well plate in a drop of 8 μL BME diluted 3 : 4 with the medium. Tumour organoids were grown for 4 days, subsequently stained with Hoechst 33342, and the DNA content was analysed on a plate reader Infinite® 200 PRO (TECAN, Männedorf, Switzerland) as well as by fluorescent microscopy (AXIO Observer.Z1 Microscope; Zeiss, Aalen, Germany) [33].

### 2.10 Lentiviral transduction of tumour organoids

Production of lentiviral vectors was performed as described [36]. In brief, Cre‐lentivirus was produced in HEK293T cells using Cre‐IRES‐PuroR plasmid (gift from D. Kotton, Addgene plasmid #30205, Watertown, MA, USA) [37] with PAX2 and VSV‐G packaging plasmids. The supernatant was passed through a 0.45 μm filter and concentrated (20 000 ***g***, 1 h, 4 °C). Virus particles were resuspended in Advanced DMEM/F12 supplemented with 10 mm HEPES, 1× Glutamax, 1× penicillin/streptomycin and stored at −80 °C. Lentiviral transduction of organoids was performed as described [36]. Briefly, organoid cells were singularized by pipetting several times and subsequent accutase digestion. After washing, cells were spinfected (500 ***g***, 1 h, 32 °C) with 8 μg·mL^−1^ polybrene. After incubation for 3 h, cells were seeded in BME and selection was started after 48 h with 1 μg·μL^−1^ puromycin.

### 2.11 Subcutaneous transplantation of tumour organoids

Subcutaneous transplantation of tumour organoids was performed as described previously [38] with minor modification. Organoids were dissociated into small cell clusters. About 2 × 10^5^ cells were resuspended in organoid basal medium, mixed with 50% BME to a final volume of 200 μL and injected subcutaneously into the flanks of C57BL/6 wild‐type mice. In some experiments, also lymphopenic Rag1^−/−^ recipient mice were used. Tumour sizes were measured using a sliding calliper and tumour volume was calculated as 0.523 × length × width × width. At day 12 or 24 after subcutaneous transplantation, mice were euthanized, subcutaneous tumours were collected, individual tumour weight and volume were measured and tumours were used for histological and flow cytometry analysis. In some experiments, wild‐type recipient mice were treated with 0.28 mg·mL^−1^ dexamethasone in the drinking water *ad libitum* as described previously [39].

### 2.12 Flow cytometry

Tumours were isolated 9 or 12 days after s.c. transplantation of tumour organoids, and digested in DMEM medium containing 10% FCS, 0.75 mg·mL^−1^ collagenase IV (Sigma‐Aldrich), 40 μg·mL^−1^ DNase I (Roche Diagnostics, Indianapolis, IN, USA) and 3 mm CaCl_2_. Digestion was stopped using 30 μL 0.5 m EDTA and cells were filtered through a 40 μm cell strainer to obtain single‐cell suspension. Dead cell staining was performed before blocking and staining steps using the Fixable Viability Dye eFluor 455UV (65‐0868‐14; eBioscience, San Diego, CA, USA). Cells were blocked afterwards with 3% bovine serum albumin (BSA) and anti‐CD16/32 TruStain FcX™ PLUS antibody (156603; BioLegend, San Diego, CA, USA), and stained using the following surface marker detecting, fluorochrome‐conjugated antibodies purchased from BioLegend: anti‐Ly‐6G‐BV605 (127639), anti‐NK‐1.1‐BV711 (108745), anti‐Ly‐6C‐BV785 (128041), anti‐CD26‐FITC (137805), anti‐XRC1‐PE (148204), anti‐CD64‐AF‐647 (139321), anti‐CD45‐AF‐700 (103128), anti‐CD11b‐PE‐Cy7 (101216); from eBioscience: anti‐CD11c‐PE‐eFluor 610 (61‐0114‐80), anti‐MHCII‐APC‐eFluor 780 (47‐5321‐80), anti‐CD3‐eFluor 506 (69‐0032‐80), anti‐CD19‐eFluor 506 (69‐0193‐80) and anti‐Siglec‐F‐BV421 (565934; BD Biosciences, Franklin Lakes, NJ, USA). Flow Cytometry experiments were performed with LSRFortessa (BD Biosciences) and data were preprocessed using flowjo software (BD Life Sciences, Ashland, OR, USA).

### 2.13 High‐dimensional FlowSOM algorithm‐guided analysis

High‐dimensional flow cytometry analysis of tumour‐infiltrating immune cells was performed according to the workflow as previously described [29, 40]. Compensation‐corrected FCS files were cleaned, preprocessed and single, live cells were manually gated after sequential doublet discrimination and exported using flowjo software (BD Life Sciences). Exported FCS files were uploaded into the rstudio environment (Posit, Boston, MA, USA) and data transformation was performed by applying the inverse hyperbolic arcsinh function with individually set cofactors. Data were further transformed with the matrixstats r package to normalize all marker expressions between values of 0 and 1 with low (1%) and high (99%) percentiles as a limit. Each FCS sample was downsampled to 10 000 cells and combined for the Uniform Manifold Approximation and Projection (UMAP) projection using the umap r package [41]. FlowSOM algorithm‐based clustering with 100 self‐organizing map codes (by default) was applied to the combined data set and meta‐clustering was performed using the consensusclusterplus package with *k* = 27 [42]. Cluster distribution was visualized with UMAP. FlowSOM clusters were manually merged and annotated to 18 identified cell populations based on their lineage marker expression profiles.

### 2.14 RNA isolation and quantitative RT‐PCR

Total RNA from murine tissues and tumours was isolated using peqGOLD TriFast™ (Peqlab, Erlangen, Germany) or RNA isolation kits (Promega, Walldorf, Germany) according to the manufacturer's instructions, and RNA was reverse transcribed. cDNA was analysed for gene expression by real‐time PCR using SYBR^®^ Green PCR Master Mix on a One‐Step‐Plus real‐time PCR machine (Applied Biosystems, Waltham, MA, USA) and normalized to β‐actin. Primers used are listed in Table S1.

### 2.15 LRH‐1 reporter activity

The murine intestinal epithelial cell line mICcl2 was transfected with LRH‐1 expression vector, an LRH‐1 luciferase reporter (mLRH‐1 5xRE) and increasing concentrations of SHP expression vector [17]. LRH‐1 transcriptional activity was measured in a luciferase reporter assay. In other experiments, mICcl2 cells were transfected with LRH‐1 and increasing concentrations of SHP expression vector and luciferase reporter construct for the promoter of LRH‐1 target genes *Cyp11a1* and *Cyp11b1* [17].

### 2.16 Co‐precipitation experiment

HEK293T cells were co‐transfected with Myc/His‐tagged β‐galactosidase or Myc/His‐tagged SHP, and GFP‐tagged LRH‐1 expression plasmid using the calcium phosphate method. Cells were then lysed in buffer A (10 mm Hepes, 10 mm KCl, pH 7.9, 1 mm DTT, universal protease inhibitor (Roche), 1% NP‐40) for 10 min at RT. Nuclei were pelleted at 20 000 ***g*** for 4 min at 4 °C, and lysed in buffer B (20 mm Hepes, 500 mm NaCl, 10% glycerol, 1 mm DTT, protease inhibitor) at 4 °C for 30 min. After centrifugation at 1700 ***g***, nucleoplasm was isolated and incubated with Ni^2+^‐sepharose beads (NiNTA; Sigma, Steinheim am Albuch, Germany) for 30 min at RT. Subsequently, beads were washed 10 times in PBS, 1% Tween, 10 mm Imidazole, pH 8.0. Protein complexes were eluted in 1× PBS, 500 mm Imidazole, pH 8.0 and analysed by western blotting using anti‐Myc‐tag and GFP‐tag antibodies.

### 2.17 Analysis of human gene expression data

Clinical data as well as gene expression (FPKM – Fragments Per Kilobase of transcript per Million) for target genes (TG) of 379 Colon Adenocarcinoma (COAD) patients with any stage (I–III excluding stage IV and patients indicated as clinical stage Not Available (NA)) of disease were downloaded from TCGA Data portal (https://portal.gdc.cancer.gov/files/4060482f‐eedf‐4959‐97f1‐f8b6c529c368, Release Date: 2018‐08‐23; Release Number 12.0). We next stratified the patients into two groups according to their TG expression (group top containing patients with high expression – as the upper quartile and the group low containing patients with lower expression – lower quartile for each of the TG: CYP11A1, HSD11B1, HSD11B2, NR0B2, or NR5A2). The Kaplan–Meier estimator provided in the R language package was used for comparing the overall survival distributions between the high versus low groups. The TCGA data and R script used to perform the Kaplan–Meier analysis are available within the Supporting Information. Lastly, the comparison of immune gene expression between the high versus low groups was performed using the nonparametric Wilcoxon's signed rank test. Gene expression levels were expressed as the log RPKM (Reads per kilobase per million) mapped reads.

### 2.18 Statistical data analysis

The statistical analysis of mouse experiments was performed using graphpad prism software (version 6, Graphpad, Boston, MA, USA). Statistical tests used were unpaired Student's *t*‐test when comparing two groups and two‐way ANOVA followed by Tukey's multiple comparison test when comparing multiple groups. The Kaplan–Meier method was used to estimate survival distribution between the groups and log‐rank tests were applied to compare survival rates.

For the analysis of human gene expression data, the Kaplan–Meier estimator provided in the R‐language package was used for comparing the overall survival distributions between the high versus low groups. Lastly, the comparison of immune gene expression between the high versus low groups was performed using the non‐parametric Wilcoxon's signed rank test.

A *P*‐value < 0.05 was set as the level of significance.

## 3 Results

### 3.1 LRH‐1 regulates inflammation‐driven intestinal tumour development

**Fig. 1:**
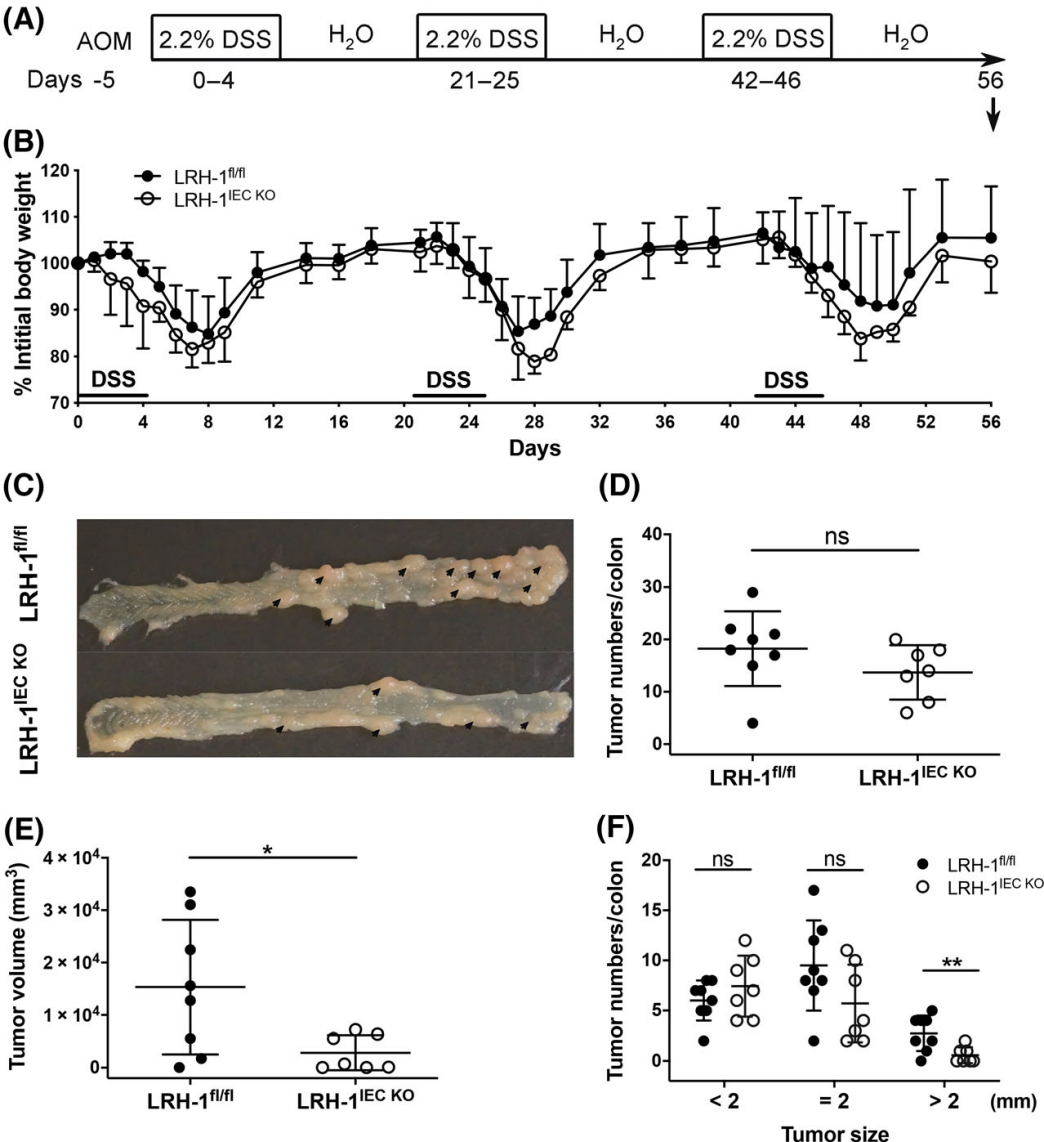
Deletion of Lrh‐1 in the intestinal epithelium attenuates colitis‐associated colorectal cancer development. (A) Scheme of azoxymethane (AOM)‐ and dextran sodium sulphate (DSS)‐induced intestinal tumour induction. (B) Weight loss curve of control (Lrh‐1^fl/fl^) mice and Lrh‐1^IEC KO^ mice after AOM treatment and during three cycles of DSS treatment. A representative experiment (*n* = 5 mice per group) of three repeats is shown (mean ± SD). (C) Colonic tumour development (arrows) in control mice (Lrh‐1^fl/fl^) and Lrh‐1^IEC KO^ mice at day 56 following AOM/DSS treatment. (D, E) Colonic tumour numbers (D) and tumour volume (E) of control and Lrh‐1^IEC KO^ mice (*n* = 7–8 per group). (F) Numbers of colonic tumours stratified according to size. (D–F) Mean ± SD of three independent experiments are shown. Unpaired Student's *t*‐test, *P < 0.05, **P < 0.01, ns: not significant. Experiments are repeated three times and data are pooled.

We and others have previously shown that LRH‐1 regulates both intestinal steroidogenesis [16, 18] as well as intestinal tumour development [25]; yet, the direct association of both processes has not been assessed so far. We, thus, set out to investigate the impact of intestinal epithelium‐specific deletion of LRH‐1 in a murine model of inflammation‐driven colorectal tumour development. A single injection of the mutagen azoxymethane (AOM), followed by three cycles of dextran sodium sulphate (DSS)‐driven intestinal inflammation (Fig. 1A) results in the robust development of colonic adenomas [43]. Treatment of mice with DSS caused an inflammation‐driven weight loss, which was restored when mice were exposed to normal drinking water [32]. In line with our previous observation that LRH‐1 regulates intestinal immune homeostasis and inflammation [25] via the synthesis of immunoregulatory glucocorticoids [16, 18, 19], we observed a substantial increase in weight loss of mice with intestine‐specific (villin‐Cre‐mediated) deletion of *Lrh‐1* (LRH‐1^IEC KO^), suggesting increased intestinal inflammation after DSS treatment (Fig. 1B). We next assessed the tumour development after three cycles of DSS. As expected at day 56 post AOM treatment, we observed smaller intestinal tumours in mice, which lacked intestinal *Lrh‐1* expression (Fig. 1C,D). While the absolute number of detectable tumours was not significantly different, LRH‐1 deficiency clearly resulted in much smaller tumours than in wild‐type mice (Fig. 1E,F). Thus, these data confirm the crucial role of LRH‐1 in regulating intestinal inflammation and tumour development [16, 19, 25].

### 3.2 SHP regulates LRH‐1 activity

**Fig. 2:**
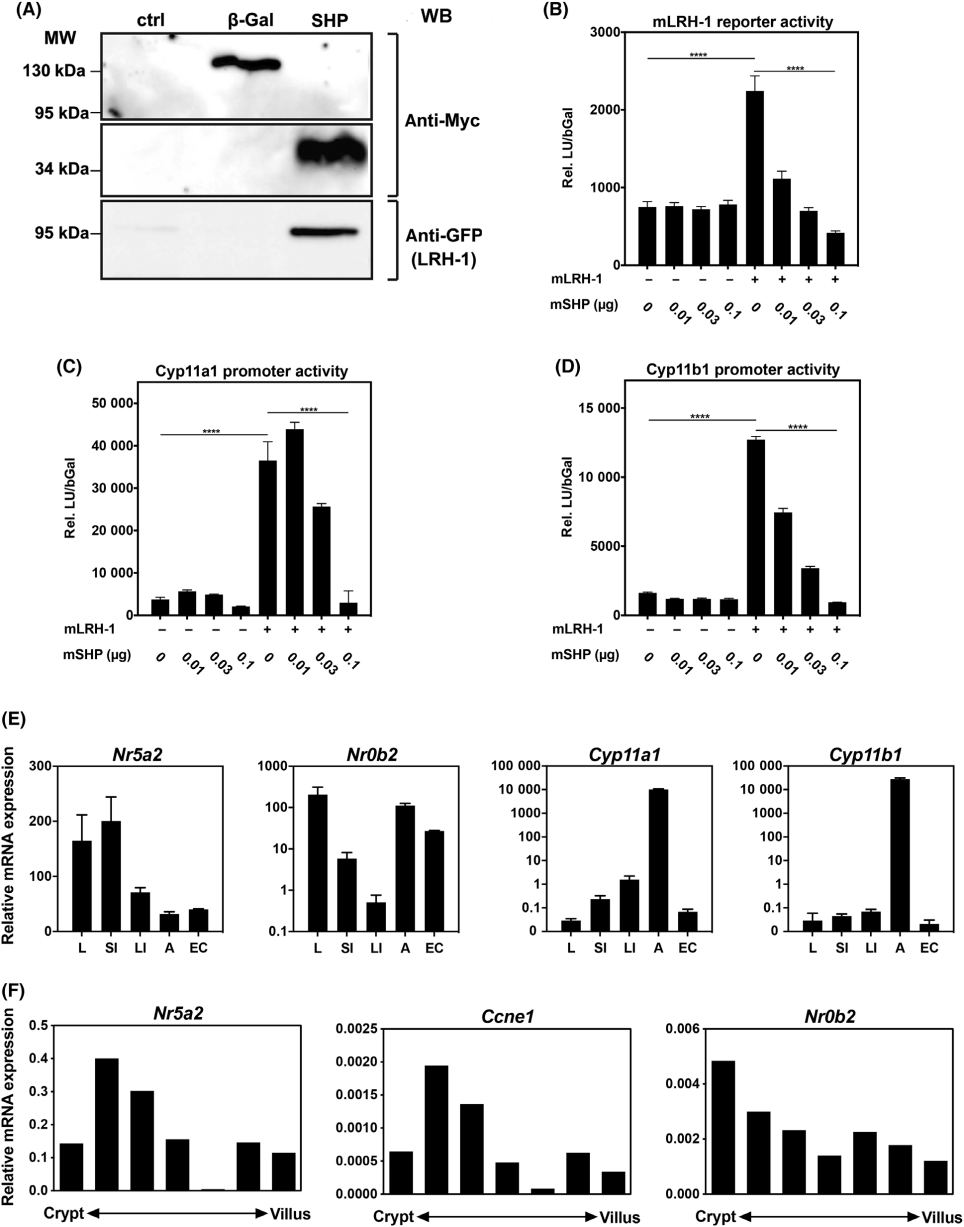
SHP suppresses LRH‐1 transcriptional activity. (A) Murine mICcl2 cells were co‐transfected with Myc/His‐tagged β‐Galactosidase (β‐Gal) as control or SHP, and GFP‐tagged LRH‐1. Nuclear lysates were precipitated with Ni^2+^‐Sepharose. Proteins were detected using anti‐Myc and anti‐GFP antibodies. (B–D) mICcl2 cells were transfected with an LRH‐1 luciferase reporter (B), Cyp11a1 promoter reporter (C) or Cyp11b1 promoter reporter (D), mLRH‐1 and increasing concentrations of mSHP expression plasmids. Relative luciferase units, normalized to β‐galactosidase activity (rel. LU/bGal) were assessed. Mean values of triplicates ± SD are shown. One‐way ANOVA with Turkey's multiple comparison ****P < 0.0001. (E) Detection of LRH‐1 (*Nr5a2*), SHP (*Nr0b2*), *Cyp11a1* and *Cyp11b1* mRNA expression by RT‐qPCR in liver, small intestine, large intestine, adrenal glands and isolated intestinal epithelial cells from wild‐type C57Bl/6 mice. Mean values of *n* = 3 ± SD are shown. (F) Expression of LRH‐1 (*Nr5a2*), cyclin E1 (*Ccne1*) and SHP (*Nr0b2*) along the crypt–villus axis. Epithelial cells from villus to crypt were differentially isolated and mRNA expression was analysed by RT‐qPCR. A typical experiment of *n* = 3 is shown.

These experiments, however, also revealed that while inflammation is essential for driving tumour development in this mouse model, LRH‐1‐regulated processes appear to be more critical in regulating tumour growth, as LRH‐1^IEC KO^ mice showed more severe chronic colitis (Fig. 1B), yet developed smaller tumours (Fig. 1E). To analyse the tumour‐promoting role of LRH‐1 in more detail, we set out to experimentally enhance LRH‐1 activity. SHP (Small Heterodimer Partner, NR0B2) is one of the important transcriptional targets of LRH‐1. At the same time, SHP binds and inhibits LRH‐1, and thus restricts LRH‐1 activity in a negative feedback loop [17, 44, 45, 46]. In pulldown experiments, we confirmed the direct physical interaction between LRH‐1 and SHP (Fig. 2A). Furthermore, SHP overexpression dose dependently inhibited LRH‐1 transcriptional activity (Fig. 2B–D). Importantly, in the small intestine, large intestine and the differentially isolated epithelial cells along the crypt–villus axis of wild‐type mice, SHP was found to be co‐expressed with LRH‐1 and the LRH‐1 target genes, the steroidogenic enzymes *Cyp11a1* and *Cyp11b1*, and *Ccne1* (cyclin E1), in the intestinal tissue and the intestinal crypts (Fig. 2E,F). Thus, intestinal SHP expression may regulate LRH‐1 activity in the intestinal epithelium.

### 3.3 SHP‐deficient mice develop less inflammation‐driven intestinal tumours

**Fig. 3:**
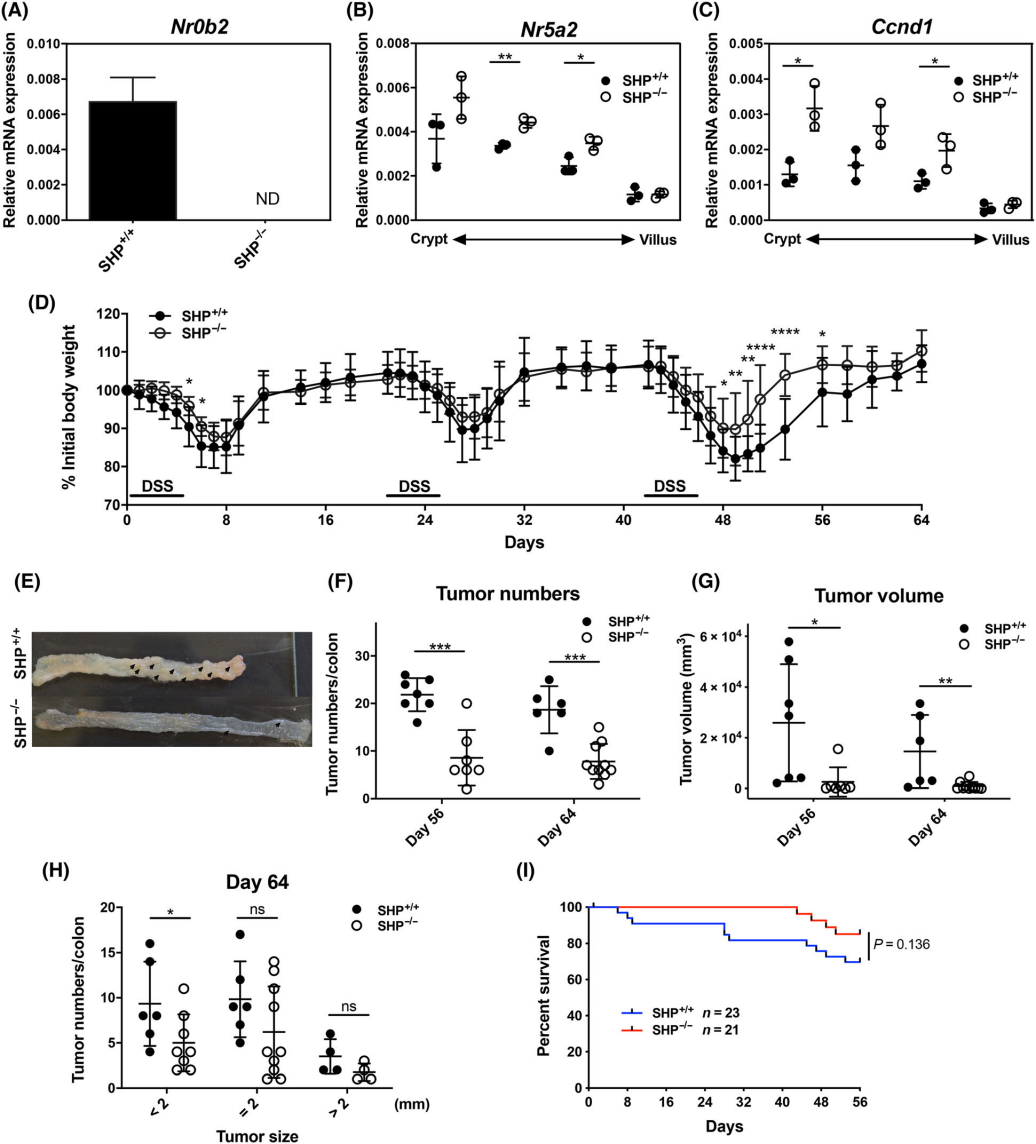
Effects of *Shp* deletion on LRH‐1‐regulated intestinal tumour development. (A) Detection of *Shp* (*Nr0b2*) in colonic tissue from control mice (Shp^+/+^) and *Shp*‐deficient mice (Shp^−/−^) (*n* = 3 mice per group, mean ± SD). ND, not detected. (B, C) Expression of Lhr‐1 (*Nr5a2*) and cyclin D1 (*Ccnd1*) along the crypt–villus axis. Epithelial cells from villus to crypt were differentially isolated from Shp^+/+^ and Shp^−/−^ mice and mRNA expression was analysed by RT‐qPCR. Mean values ± SD of three mice per group are shown. (D) Weight loss curve of control (Shp^+/+^) and Shp^−/−^ after AOM/DSS treatment. Mean values ± SD of pooled three independent experiments are shown (*n* = 7–14 mice per group). (E) Colonic tumour development (arrows) in control (Shp^+/+^) and Shp^−/−^ at day 64. (F, G) Tumour numbers (F) and tumour volume (G) of Shp^+/+^ and Shp^−/−^ mice at days 56 and 64 (mean ± SD of *n* = 7 mice per group). (H) Numbers of colonic tumours stratified according to size. Unpaired Student's *t*‐test, *P < 0.05, **P < 0.01, ***P < 0.001, ****P < 0.0001. (I) Overall survival of Shp^+/+^ and Shp^−/−^ mice during AOM/DSS‐induced colitis.

We next assessed whether genetic deletion of SHP would unleash the oncogenic activity of LRH‐1 resulting in increased AOM/DSS‐induced intestinal tumours. We first confirmed that SHP expression was undetectable in the intestine of SHP (*Nr0b2*)‐deficient mice (Fig. 3A) and that SHP deficiency resulted in increased LRH‐1 transcriptional activity. While *Lrh‐1* (*Nr5a2*) expression was only slightly increased (Fig. 3B), the expression of the LRH‐1 target gene cyclin D1 (*Ccnd1*) [22] was up to three times higher in the crypts of SHP‐deficient mice (Fig. 3C), supporting the idea that increased LRH‐1 activity results in increased target gene expression. When SHP‐deficient mice were exposed to AOM and three cycles of DSS, reduced inflammation‐driven weight loss was recorded (Fig. 3D), indicating that SHP deficiency was able to unleash the anti‐inflammatory activity of LRH‐1. Surprisingly, however, when tumour development and growth were assessed at days 56 and 64 post‐AOM/DSS treatment, a significantly reduced number and size of colorectal tumours was noticed in SHP‐deficient animals (Fig. 3E–H). Reduced tumour growth correlated also with increased survival of SHP‐deficient mice compared to wild‐type mice (Fig. 3I).

### 3.4 SHP deficiency ameliorates acute DSS‐induced colitis

**Fig. 4:**
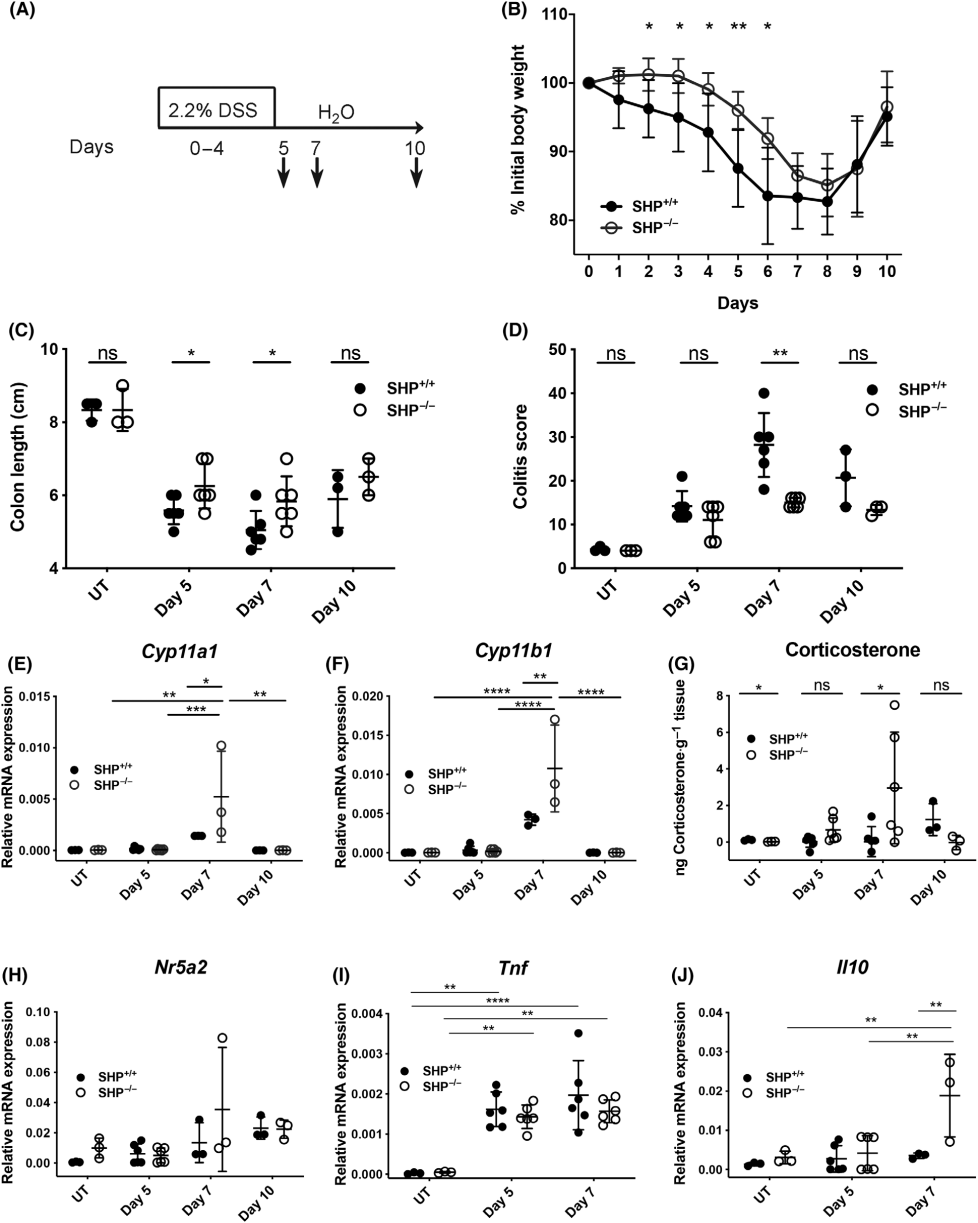
Effect of Shp on acute colitis and intestinal glucocorticoid synthesis. (A) Scheme of acute colitis induction. (B) Weight loss curve of Shp^+/+^ and Shp^−/−^ mice during acute DSS colitis (*n* = 3–6 mice per group and time point. Mean values ± SD). (C, D) Colon shortening (C) and colitis score (D) of Shp^+/+^ and Shp^−/−^ mice at days 0 (UT), 5, 7 and 10 post‐DSS treatment (*n* = 3–6 mice per group and time point. Mean values ± SD). (E, F) Colonic *Cyp11a1* (E) and *Cyp11b1* (F) mRNA expression in Shp^+/+^ and Shp^−/−^ mice at day 0 (UT), days 5, 7 and 10 post‐DSS treatment (*n* = 3–6 mice per group and time point. Mean values ± SD). (G) *Ex vivo* colonic corticosterone synthesis (ng·g^−1^ tissue) in Shp^+/+^ and Shp^−/−^ mice at day 0 (UT), days 5, 7 and 10 post‐DSS treatment (*n* = 3–6 mice per group and time point. Mean values ± SD). (H–J) Colonic *Nr5a2* (H), *Tnf* (I) and *Il10* (J) mRNA expression in Shp^+/+^ and Shp^−/−^ mice at day 0 (UT), days 5 and 7 post‐DSS treatment (*n* = 3–6 mice per group and time point. Mean values ± SD). Unpaired Student's *t*‐test (B–D, G), two‐way ANOVA followed by Tukey's multiple comparison test (E, F, H–J), *P < 0.05, **P < 0.01, ***P < 0.001, ****P < 0.0001, ns: not significant. Typical experiments of *n* = 3 are shown.

Since AOM/DSS‐induced tumour development strongly depends on chronic intestinal inflammation, we investigated whether reduced inflammation in SHP‐deficient mice could be responsible for the reduced development of intestinal tumours. Indeed, when SHP‐deficient mice were exposed to acute DSS‐induced colitis (Fig. 4A), a significant delay in weight loss was noticed (Fig. 4B). This was accompanied by reduced inflammation‐driven colon shortening (Fig. 4C) and colitis score (Fig. 4D). In line with the increased activity of LRH‐1 in SHP‐deficient mice, we observed increased colitis‐induced expression of the steroidogenic enzymes *Cyp11a1* and *Cyp11b1* (Fig. 4E,F), and associated production of colonic corticosterone (Fig. 4G). In parallel, a significant increase of the immunosuppressive cytokine interleukin (IL)‐10 and a trend towards reduced tumour necrosis factor (TNF) expression was noticed in SHP‐deficient mice at the peak of DSS colitis at day 7, while LRH‐1 expression was not different (Fig. 4H–J). Thus, SHP deficiency is associated with reduced DSS‐induced inflammation, and increased immunoregulatory corticosterone synthesis and IL‐10 expression.

### 3.5 Intestinal glucocorticoid synthesis regulates tumour development and growth

We next aimed at more specifically investigating the relative contribution of intestinal steroidogenesis in the development of inflammation‐driven intestinal tumour development. For this purpose, we developed a novel mouse model with defective intestinal glucocorticoid synthesis. 11β‐hydroxylase, encoded by the gene *Cyp11b1*, converts the inactive precursor molecule deoxycorticosterone to the active glucocorticoid corticosterone. We, thus, generated a mouse line, where exons 3–5 of the *Cyp11b1* gene were deleted in the intestinal epithelium using villin promoter‐driven Cre recombinase expression (Cyp11b1^IEC KO^) (Fig. S1A–C). As expected, the intestinal epithelium of these conditional knockout mice showed a drastically reduced expression of *Cyp11b1* (Fig. S1D) and immune cell‐driven corticosterone production (Fig. S1F,G), while the adrenal synthesis of glucocorticoids remained unaffected (Fig. S1E). These data confirm the intestine‐specific deletion of *Cyp11b1* and associated loss of intestinal glucocorticoid synthesis.

**Fig. 5:**
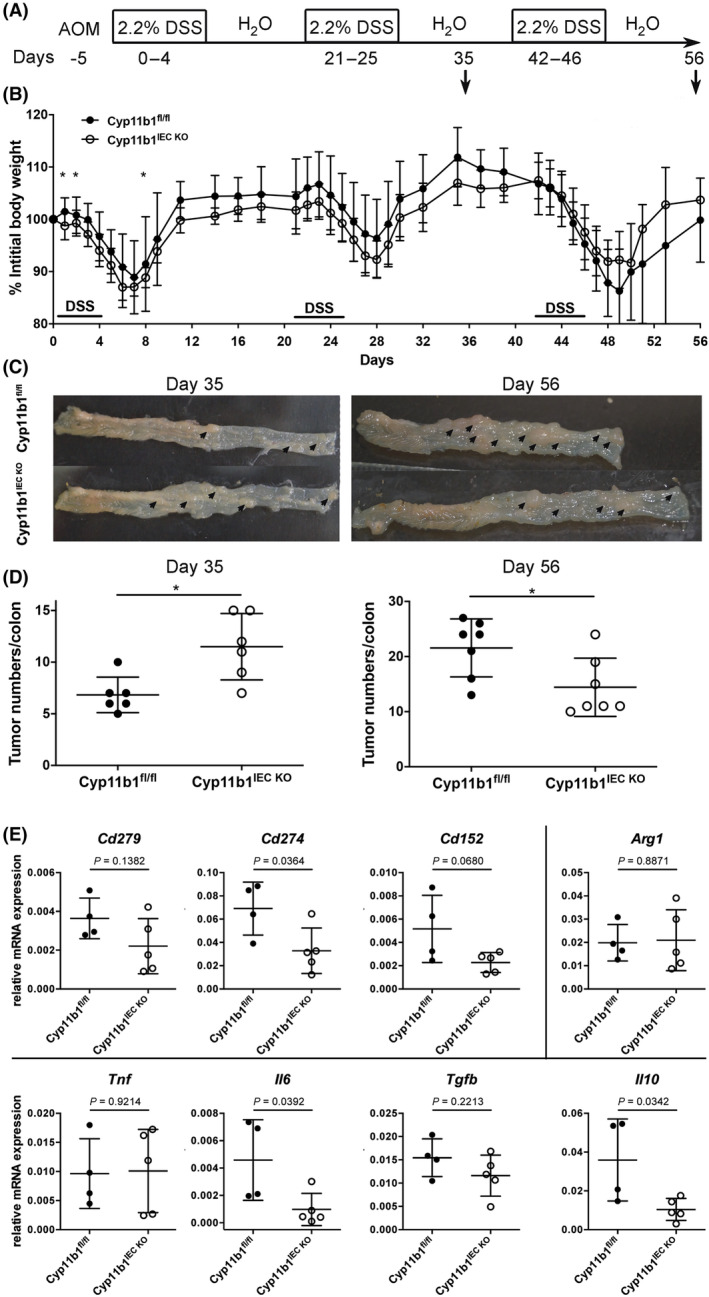
Role of colonic glucocorticoid synthesis in intestinal tumour development. (A) Scheme of AOM/DSS‐induced intestinal tumour induction. (B) Weight loss curve of control (Cyp11b1^fl/fl^) and Cyp11b1^IEC KO^ mice after AOM/DSS treatment. Mean values ± SD of pooled three independent experiments are shown (*n* = 6–13 mice per group). Unpaired Student's *t*‐test, *P < 0.05. (C) Colonic tumour development (arrows) in Cyp11b1^fl/fl^ and Cyp11b1^IEC KO^ mice at days 35 and 56. (D) Colonic tumour numbers in Cyp11b1^fl/fl^ and Cyp11b1^IEC KO^ mice at days 35 and 56 (*n* = 6–7 mice per group and time point). Unpaired Student's *t*‐test, *P < 0.05. (E) Detection of immune checkpoints (*Cd279*, *Cd274*, *Cd152*), M2 macrophage marker (*Arg1*) and cytokines (*Tnf*, *IL6*, *Tgfb*, *Il10*) in Cyp11b1^fl/fl^ and Cyp11b1^IEC KO^ tumours at day 48 (*n* = 4–5 mice). Unpaired Student's *t*‐test, *P*‐values are indicated.

We next induced intestinal tumour development in these mice by AOM injection and chronic DSS colitis (Fig. 5A). Interestingly, while Cyp11b1^IEC KO^ mice showed significantly increase body weight loss in the first two cycles of DSS, suggesting increased intestinal inflammation in the absence of immunoregulatory glucocorticoids, Cyp11b1^IEC KO^ mice recovered faster after the third cycle of DSS (Fig. 5B). In line with increased inflammation, we also observed an increase in colonic tumours in Cyp11b1^IEC KO^ mice at day 35 after two cycles of DSS, but a significant reduction in tumour numbers at day 56 (Fig. 5C,D). Cyp11b1‐deficient mice not only showed reduced tumour numbers, but tumours were also smaller. Furthermore, Cyp11b1‐deficient tumours had reduced expression of the immunoregulatory molecules CD274 (PD‐L1), CD152 (CTLA‐4) and IL‐10 (Fig. 5E). This observation likely reflects the immunosuppressive effects of glucocorticoids released from already established tumours, enabling immune escape.

### 3.6 Transplantation of 3D tumoroids reveals the oncogenic potential of LRH‐1

**Fig. 6:**
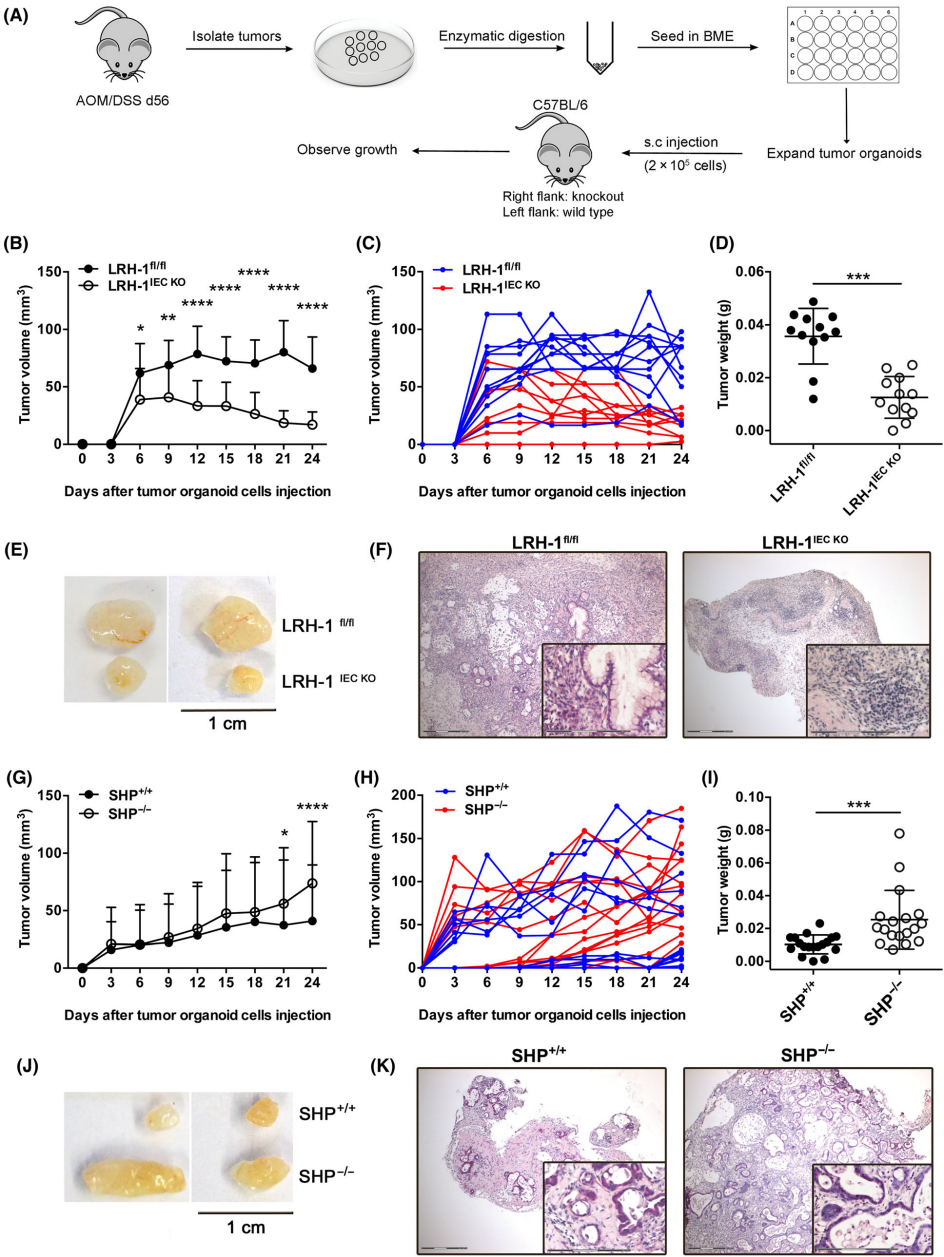
Role of LRH‐1 and Shp in growth regulation of transplanted tumour organoids. (A) Scheme of tumour organoid development and s.c. transplantation into C57Bl/6 wild‐type recipients. (B) *In vivo* growth of transplanted control (Lrh‐1^fl/fl^) and Lrh‐1^IEC KO^ tumour organoids after s.c. transplantation into C57Bl/6 wild‐type mice. Mean values ± SD of *n* = 12 mice per group are shown. Experiments are repeated twice. (C) Growth of individual tumours is summarized in B. (D) Weight of isolated tumours from the experiment shown in B. (E) Examples of tumours isolated at day 24 post transplantation. (F) Histology of Lrh‐1^fl/fl^ and Lrh‐1^IEC KO^ tumours at day 24 post transplantation. The inlay shows magnification. Scale bars: overview 300 μm, inlay 150 μm. (G) *In vivo* growth of transplanted control Shp^+/+^ and Shp^−/−^ tumour organoids after s.c. transplantation into C57Bl/6 wild‐type mice. Mean values ± SD of *n* = 12 mice per group are shown. Experiments are repeated twice. (H) Growth of individual tumours summarized in G. (I) Weight of isolated tumours from the experiment shown in G. (J) Examples of tumours isolated at day 24 post transplantation. (K) Histology of control Shp^+/+^ and Shp^−/−^ tumours at day 24 post transplantation. The inlay shows magnification. Scale bars: overview 300 μm, inlay 150 μm. Mean ± SD is shown. Unpaired Student's *t*‐test, *P < 0.05, **P < 0.01, ***P < 0.001, ****P < 0.0001.

These experiments described above demonstrated that LRH‐1 and intestinal glucocorticoid synthesis may affect inflammation‐driven intestinal tumorigenesis at two different levels. While LRH‐1 is critical for tumour growth via the transcriptional regulation of cell cycle‐regulating genes, it also controls inflammation via the regulation of glucocorticoid synthesis in the normal intestinal epithelium, thereby limiting colitis‐induced tumour development. On the other hand, tumour‐expressed LRH‐1 and associated glucocorticoid synthesis by intestinal tumours may favour immune escape and thus promote tumour development and growth. To analyse these processes independently of the initial tumour‐promoting inflammatory phase during the chronic colitis, we isolated tumours from wild‐type mice, LRH‐1‐deficient or SHP‐deficient mice at day 56, and expanded tumour cells in 3D cultures resulting in tumour organoids (tumoroids) (Fig. 6A, Fig. S2A). Although LRH‐1 regulates cell growth, wild‐type, LRH‐1‐deficient as well as SHP‐deficient tumoroids could be expanded *in vitro* (Fig. S2B–E). When tumoroids were sufficiently expanded, they were transplanted s.c. into wild‐type immunocompetent C57Bl/6 mice. Importantly, each recipient mouse was simultaneously transplanted with wild‐type tumoroids and LRH‐1‐, resp. SHP‐deficient tumoroids, to directly compare tumour growth. Tumoroids grew very rapidly to a palpable size at days 6–12 post transplantation. In line with the oncogenic role of LRH‐1, a significantly reduced growth was observed for LRH‐1‐deficient tumoroids (Fig. 6B–E). Interestingly, while in transplanted wild‐type tumoroids, tumour cells were interspersed in tumour stroma, almost no LRH‐1‐deficient tumour cells were detectable anymore at day 12. In contrast, increased immune cell infiltrates were evident (Fig. 6F). As expected, the opposite phenotype was observed when SHP‐deficient tumoroids were transplanted. In line with unleashed LRH‐1 activity, they grew significantly faster and bigger than wild‐type tumoroids (Fig. 6G–K).

### 3.7 Tumour‐derived glucocorticoids favour tumour growth and immune escape

**Fig. 7:**
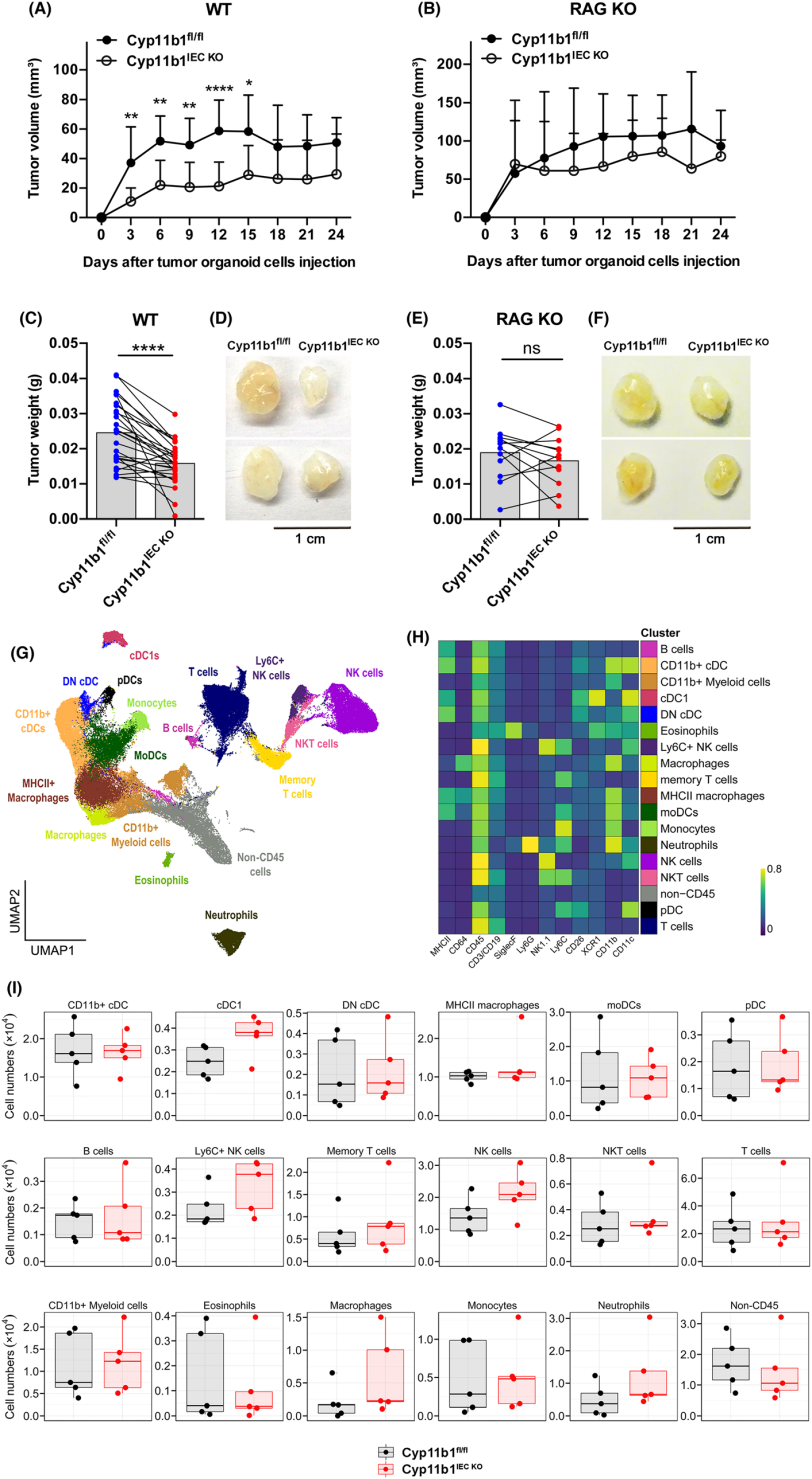
Role of tumour glucocorticoid synthesis in immune evasion of transplanted tumour organoids. Control (Cyp11b1^fl/fl^) and Cyp11b1^IEC KO^ tumour organoids were transplanted into immunocompetent C57Bl/6 wild‐type recipients (WT) (A) or lymphopenic *Rag1*‐deficient mice (RAG KO) (B). (A, B) Tumour growth of transplanted tumours. Mean values ± SD of *n* = 12–26 mice per group are shown. (C, E) Mean values ± SD of tumour weight of isolated Cyp11b1^fl/fl^ and Cyp11b1^IEC KO^ tumours at day 12 post transplantation into wild‐type recipient mice (C) (*n* = 26 mice per group) or *Rag1*‐deficient mice (E) (*n* = 12 mice per group). Data points of tumours from the same recipient mice have been connected by lines. Unpaired Student's *t*‐test. ns not significant, *P < 0.05, **P < 0.01, ***P < 0.001, ****P < 0.0001. Experiments have been repeated four times (A) and two times (B). (D, F) Examples of tumours isolated at day 12 post transplantation. (G–I) High‐dimensional flow cytometry analysis of tumour‐infiltrating immune cells from control (Cyp11b1^fl/fl^)‐ or *Cyp11b1*‐deficient (Cyp11b1^IEC KO^) tumours at day 9 post transplantation. Data represent combined samples with *n* = 5 mice per group. (G) UMAP plot with manually annotated FlowSOM cell clusters of 100 000 cells combined from all tumours (10 000 cells randomly sampled per tumour). (H) Heatmap with lineage marker expressions (columns) over the manually annotated FlowSOM cell clusters (rows). (I) Total cell numbers of indicated cell clusters are presented as box plots showing the 25th to 75th percentiles with whiskers indicating the range to the smallest and largest data point until the 1.5× IQR. Dots in box plots represent individual mice. Box plots with median values and lower and upper quartiles are shown. DN cDCs, double‐negative conventional dendritic cells; moDCs, monocyte‐derived dendritic cells; lineage, pDCs, plasmacytoid dendritic cells.

We next aimed to address the relative contribution of LRH‐1‐regulated tumour‐autonomous glucocorticoid synthesis in the expansion of transplanted tumours. We, thus, first confirmed that wild‐type tumoroids produce glucocorticoids, whereas Cyp11b1‐deficient tumoroids produce less (Fig. S2F). Strikingly, while control tumoroids expanded rapidly after transplantation, a significant delay was monitored in Cyp11b1^IEC KO^ tumoroid transplants (Fig. 7A,D). This was also reflected by the weight of the tumours at day 12 post transplantation (Fig. 7C). Importantly, no difference in control or Cyp11b1^IEC KO^ tumoroid growth was observed *in vitro* (Fig. S2C). These data support the notion that tumours incapable of releasing immunoregulatory glucocorticoids are controlled more efficiently by the immune system. To provide further evidence for the hypothesis that glucocorticoids mediate tumour immune evasion, wild‐type recipient mice were control treated or received the synthetic glucocorticoid dexamethasone in the drinking water. Notably, while in untreated recipient mice, Cyp11b1‐proficient tumours grew faster and bigger than Cyp11b1‐deficient ones, no statistical difference was observed anymore in dexamethasone‐treated recipient mice (Fig. S3A). Similarly, when Cyp11b1 was deleted *in vitro* by transducing Cyp11b1L/L tumour organoids with lentiviral Cre recombinase, a similar growth advantage of Cyp11b1‐proficient tumours over Cyp11b1‐deleted tumours was observed upon s.c. transplantation (Fig. S3B–E). These findings support the idea that lack of tumour glucocorticoid synthesis, rather than differences in genetic mutations, is responsible for the reduced growth observed. We next transplanted Cyp11b1‐proficient and Cyp11b1‐deficient tumoroids into lymphopenic Rag1‐deficient mice to monitor the contribution of T and B cells in the control of tumour growth. Interestingly, while we could still see enhanced growth of control tumoroids, differences to Cyp11b1‐deficient tumoroids were less pronounced than when transplanted into wild‐type recipients (Fig. 7B). Importantly, when tumour sizes were analysed at day 12 post transplantation, no significant difference was observed (Fig. 7E,F). These findings suggest a contribution of immune cells, in part also lymphocytes, in the control of tumour growth, which is suppressed by tumour‐derived glucocorticoids.

In support of this notion, we detected reduced expression of the M2‐like macrophage marker arginase 1 (*Arg1*), but increased expression of the pro‐inflammatory cytokine IL‐6 in Cyp11b1‐deficient transplanted tumoroids. While IL‐10 expression was not significantly different, there was a tendency towards lower expression in Cyp11b1‐deficient tumours (*P* = 0.0837) (Fig. S4A). These changes were paralleled by a significant increase in CD45^+^ and CD11b^+^ leukocytes, while levels of CD3^+^ and FoxP3^+^ T_reg_ were not significantly different (Fig. S4B). To further characterize the differences in tumour‐infiltrating immune cells, we employed high‐dimensional flow cytometry with computational clustering and analysis of immune cell populations (Fig. S4C, Fig. 7G–I). These analyses of immune cell infiltrates in Cyp11b1‐proficient and Cyp11b1‐deficient transplanted tumours revealed substantial changes in innate immune cell populations, especially dendritic cells, macrophages, natural killer (NK) cells and neutrophils at day 9 post transplantation (Fig. 7I).

### 3.8 Survival of colorectal tumour patients negatively correlates with the expression of steroidogenic factors and immunomodulatory markers

**Fig. 8:**
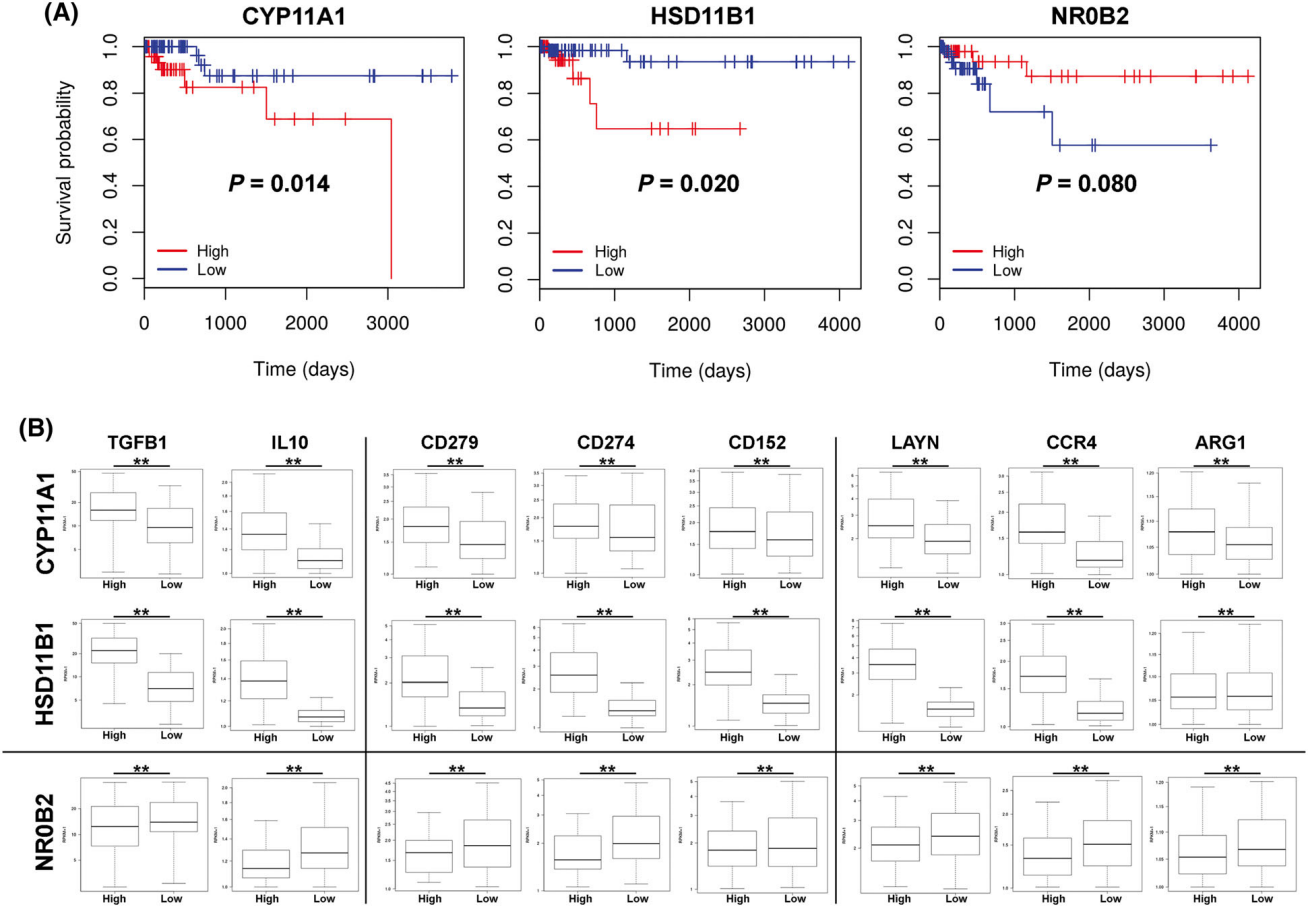
Survival curves of colon cancer patients with high or low expression of *CYP11A1*, *HSD11B1* and *NR0B2*. (A) Colon cancer patients were stratified into groups of the highest or lowest quartile expression of the genes *CYP11A1*, *HSD11B1* or *NR0B2* (SHP), and survival was analysed (95 patients per group). Statistical differences (*P‐*values) are indicated. (B) Individual tumours from the highest or lowest quartile expression of *CYP11A1*, *HSD11B1* and *NR0B2* were analysed for the expression of genes related to immunoregulatory cytokines (*TGFB1*, *IL10*), immune checkpoints (*CD279* (PD‐1), *CD274* (PD1‐1L), *CD152* (CTLA‐4)), and M2‐like macrophage markers (*LAYN*, *CCR4*, *ARG1*). The data were derived from 379 colon adenocarcinoma (COAD) samples from the TCGA database using upper (high) quartile (*n* = 95) versus lower (low) quartile (*n* = 95) expression of the given gene (*CYP11A1*, *HSD11B1* or *NR0B2*) to form the groups analysed. Kaplan–Meier survival plots comparing the higher quartile versus lower quartile groups defined by expression of each gene indicated. The expression levels of immune genes are shown as the log RPKM – reads per kilobase per million mapped reads. Box plots with median values, lower and upper quartiles and minima and maxima are shown. All markers show significant differences (**P < 0.01) between high and low *CYP11A1*, *HSD11B1* and *NR0B2* expressors.

In order to assess the potential relevance of our findings for the pathogenesis of human colorectal cancer, we analysed the expression profile of *CYP11A1*, *HSD11B1*, *HSD11B2*, *NR5A2* (LRH‐1) and *NR0B2* (SHP) in tumours from 379 colorectal cancer patients, and correlated their overall survival to the expression of these steroidogenic factors. CYP11A1 (p450 side chain‐cleaving enzyme) is the rate‐limiting and thus essential enzyme in the *de novo* synthesis of glucocorticoids from cholesterol, whereas HSD11B2 (hydroxysteroid dehydrogenase 11B2) deactivates cortisol to cortisone, and HSD11B1 reactivates cortisone to cortisol and thus contributes to the regulation of local glucocorticoid levels (reviewed in Ref. [13, 26]). When patients were stratified into high expressors (highest quartile) and low expressors (lowest quartile), a significant difference in patient overall survival could be seen for *CYP11A1*, *HSD11B1* and *NR0B2* (Fig. 8A), but not for *NR5A2* and *HSD11B2* (Fig. S5A). Interestingly, while high *CYP11A1* and *HSD11B1* expression resulted in poor survival, suggesting that tumours efficiently suppressing the immune system via glucocorticoids have a growth advantage, high *NR0B2* was beneficial for survival, likely due to increased suppression of LRH‐1. The proposed role of tumour glucocorticoid synthesis in suppressing anti‐tumour immune responses was further supported by the observation that high *CYP11A1* and *HSD11B1* expression also resulted in a significantly increased expression of the immunosuppressive cytokines TGFβ1 and IL‐10, the immune checkpoint molecules CD279 (PD‐1), CD274 (PD‐L1) and CD152 (CTLA‐4), and the M2‐like macrophage markers LAYN, CCR4 and ARG1 [47]. In contrast, high *NR0B2* expression correlated with a significantly lower expression of these immune markers (Fig. 8B). These findings support the hypothesis that glucocorticoid synthesis in colorectal tumours results in a suppressive tumour microenvironment, favouring the escape of the tumour from destruction by the immune system and worse patient survival.

## 4 Discussion

It is currently well accepted that the immune system critically regulates tumour development. A limiting aspect in tumour immune surveillance, however, are all the factors that control self‐tolerance and thus limit the activation of tumour‐specific immune cells. Currently best studied are the immune checkpoint molecules PD‐1/PD‐L1 and CLTA‐4. The recognition of the importance of these immune checkpoints for controlling the immune system in general, and anti‐tumour responses in particular, has led to the development of neutralizing antibodies against these checkpoint molecules and their successful use as therapeutic agents. The results in the treatment of some but not all tumours are astonishing, in particular in combination with conventional chemotherapy. Yet, the unsuccessful treatment of many patients clearly indicates that other potential immune checkpoints may exist.

Here, we provide evidence that tumour‐derived glucocorticoids may indeed represent such a novel “checkpoint”, and that inhibition of tumour glucocorticoid synthesis could develop as an effective therapeutic approach. Although we transplanted syngeneic tumours into syngeneic recipient mice, in the absence of dominant tumour model antigens, our data point out that inhibition of glucocorticoid synthesis in tumours alone is sufficient to promote a protective immune response, causing a strong reduction in tumour growth. Importantly, differences in genetic mutations of the transplanted tumours could be excluded as *in vitro* deletion of the *Cyp11b1* gene in tumour organoids by lentiviral Cre transduction resulted in similar reduced tumour growth upon s.c. transplantation. To what extent this immune surveillance is mediated by tumour‐specific T lymphocytes, innate immune cells, such as NK cells and neutrophils, or a combination thereof [48, 49, 50], remains to be determined. Clearly, Cyp11b1‐deficient tumours still grew slower, even in T‐cell‐depleted Rag‐deficient mice (Fig. 7B), and had increased numbers of infiltrating macrophages, dendritic cells, neutrophils and NK cells (Fig. 7I). Thus, it is very likely that both cells of the adaptive and innate immune system contribute to the control of tumour growth. Furthermore, the relative contribution of cells of the innate and adaptive immune system may be different during the development of primary, inflammation‐driven intestinal tumours versus the transplantation of tumour organoids.

Excitingly, our experimental data in mice also strongly correlated with the gene expression and survival data in human colorectal cancer patients. The expression levels of the two steroidogenic enzymes CYP11A1, promoting *de novo* synthesis of glucocorticoids from cholesterol, and HSD11B1, initiating reactivation of glucocorticoids from inactive precursors (reviewed in Ref. [12, 13]), significantly correlated with the overall patients' survival (Fig. 8A). In marked contrast, high expression of SHP, likely by suppressing LRH‐1 and associated proliferation and steroidogenesis, positively correlated with patient survival. These data indeed suggest that glucocorticoid synthesis in colorectal tumour patients actively suppresses tumour surveillance by the immune system. In support of this idea, it is also our observation that high expression of the steroidogenic enzymes CYP11A1 and HSD11B1 correlated with high expression of the immunosuppressive cytokines TGFβ and IL‐10, the immune checkpoint molecules PD‐1, PD‐L1 and CTLA‐4 and the presence of LAYN^+^ and CCR4^+^ tumour‐associated M2‐like macrophages (Fig. 8B). A similar pattern of immune markers was also observed in primary mouse tumours (Fig. 5E). In support of this idea, it was recently observed that glucocorticoids upregulate immune checkpoint molecules in the tumour microenvironment [51, 52]. Thus, tumour‐autonomous glucocorticoid synthesis appears to substantially condition the tumour microenvironment, ultimately suppressing anti‐tumour immune responses and resulting in decreased patient survival. Somewhat surprising was the observation that expression levels of LRH‐1 did not significantly affect patients' survival. While the underlying reason is difficult to reconcile, it has to be considered that LRH‐1 is involved in the transcriptional control of a broad spectrum of processes, including proliferation, survival, metabolism and steroidogenesis, which may have even conflicting effects on tumour development. In contrast, expression levels of steroidogenic enzymes represent only one spectrum of LRH‐1 target genes and will, thus, affect only the steroidogenic capacity of colorectal tumours.

A major aim of this study has been to investigate the relative contribution of LRH‐1‐regulated glucocorticoid synthesis in the inflammation‐induced development of colorectal tumours. Our results point out that intestinal glucocorticoid synthesis has a prominent dual role in this process. At the early phase, LRH‐1‐regulated intestinal glucocorticoid synthesis appears to suppress intestinal inflammation and thus associated tumour development. Along these lines, we have seen that intestine‐specific deletion of *Cyp11b1*, abrogating intestinal glucocorticoid synthesis, resulted in increased inflammation and increased tumour development during the early phases of chronic colitis (Fig. 5B–D). In contrast, the deletion of SHP resulted in increased LRH‐1‐regulated steroidogenesis and reduced inflammation‐driven tumour development. Thus, the deletion of SHP was able to unleash the anti‐inflammatory activity of LRH‐1, rather than its oncogenic potential. Of interest is the observation that although deletion of LRH‐1 resulted in increased inflammation, rather reduced tumour development was observed. This illustrates that increased inflammation *per se* is insufficient to promote tumour development, but that the proliferation‐promoting activity and likely also the glucocorticoid‐promoting activity of LRH‐1 in already established tumours is necessary for efficient tumour growth and survival. In support of this hypothesis, we have seen that in the absence of an inflammatory environment (i.e. in the transplantation experiment) deletion of SHP was able to promote the oncogenic activity of LRH‐1, resulting in increased growth of transplanted tumoroids (Fig. 6G–K). While these experiments do not reveal the relative importance of LRH‐1 in regulating tumour growth via the transcriptional control of cell cycle‐regulating genes versus the suppression of anti‐tumour immune responses via tumour‐derived glucocorticoids, the transplantation of Cyp11b1‐deficient tumoroids, which maintain‐LRH‐1‐regulated proliferation, clearly support the idea that the local synthesis of glucocorticoids critically contributes to the evasion of colorectal tumours from tumour immune surveillance, and thus promotes their survival and growth. In line with this concept, it is our observation that although Cyp11b1‐deficient mice showed increased tumour development during the early phase of chronic colitis (day 35) due to increased inflammation‐driven tumour development, the opposite was seen after the third cycle of DSS (day 56) (Fig. 5C,D), indicating that in already established tumours, glucocorticoid‐mediated immune evasion is essential for their survival. Furthermore, we have seen that treatment of recipient mice with dexamethasone abolished the growth differences between Cyp11b1‐proficient and deficient tumoroids after transplantation (Fig. S3).

While the oncogenic role of LRH‐1 has been well documented in a variety of tumours, the role of steroidogenic enzymes in the development of tumours, and colorectal tumours in particular, is less clear. In colon cancer, *CYP11A1* appears to be frequently downregulated [53]. Nonetheless, our own data demonstrate that high *CYP11A1* expression results in increased tumour growth and reduced patient survival. Interestingly, certain *CYP11A1* polymorphisms have also been associated with an increased risk to develop breast cancer. In addition, *CYP11B1*, encoding 11β‐hydroxylase, has been reported to be frequently mutated in different tumours, including colorectal tumours [53]. Although the impact of these mutations on the expression and function of this enzyme has not been studied so far, it is very likely that they are part of a selection process during tumour development. Of interest, while we have seen significant differences in overall patient survival between tumours with high versus low *CYP11A1* expression (Fig. 8A), we have not been able to conduct the same analysis for *CYP11B1*, as expression was not reported for all tumour tissues summarized in the TCGA database. One of the underlying reasons may well be that basal expression levels of *CYP11B1* may be lower than those of *CYP11A1*. This notion is in line with our previous observation in the murine intestine, where *Cyp11b1* was barely detectable in control mice, but strongly upregulated upon immune cell activation or by inflammation (Fig. S1D) [18, 32, 54]. Most importantly, 11β‐hydroxylase appears to be essential for glucocorticoid synthesis in the intestinal epithelium and intestinal tumours, as *Cyp11b1* deletion abrogated it (Fig. S1F,G).

Based on the here discussed role of tumour‐derived glucocorticoids in immune escape, LRH‐1‐regulated steroid synthesis in tumour cells may represent an attractive new therapeutic approach. However, a general suppression of glucocorticoid synthesis will likely result in severe side effects, given the widespread roles of glucocorticoids in numerous physiological processes. Thus, a detailed understanding of the tumour‐specific processes controlling glucocorticoid synthesis in colorectal cancer may be instrumental to develop tumour‐specific therapies. In this regard, LRH‐1 seems to be the most promising target.

## 5 Conclusions

We have previously shown that human colorectal tumour cells are capable of producing immunoregulatory glucocorticoids in an LRH‐1‐regulated manner. In this study, we now specifically addressed how tumour‐derived glucocorticoids regulate anti‐tumour immune responses and tumour growth in a murine model of inflammation‐driven colorectal tumour development. Our results show that tumours lacking LRH‐1 expression or its transcriptional target Cyp11b1 fail to produce tumour‐autonomous glucocorticoids, which results in better control of the tumour growth by tumour‐infiltrating immune cells. Thus, tumour‐derived glucocorticoids appear to be an important immune checkpoint. Comparative gene expression and survival data analysis indicate that similar immune escape mechanisms exist also in human colorectal cancer patients.

## Conflict of interest

The authors declare no conflict of interest.

## Author contributions

AA planned and conducted most experiments, and generated the figures; CR, EB, CU, MN, TSP, LD, VMM, FK and FN conducted some experiments; FK and RS characterized the Cyp11b1^IEC KO^ mouse model; JK helped with the AOM/DSS tumour model; KB, CM and HFF helped with the tumoroid system; APP contributed to the figure generation; ITS and KJG analysed the gene expression databases; EK advised on clinical samples; TB supervised the study and wrote the manuscript.

## Supporting information


**Fig. S1.** Generation and characterization of intestine‐specific Cyp11b1‐deficient mice.
**Fig. S2.** *In vitro* growth of tumour organoids.
**Fig. S3.** Effect of dexamethasone treatment or *Cyp11b1* deletion on *in vivo* growth of s.c. transplanted tumours.
**Fig. S4.** Characterization of tumour immune infiltrates.
**Fig. S5.** Effect of NR5A2 and HSD11B2 expression on colorectal patient survival and immune marker expression.

**Table S1.** List of PCR and qRT‐PCR primers.

## Data Availability

The data sets generated and/or analysed during the current study are available from the corresponding authors upon reasonable request.
