# Supplementary material for: Immune escape of colorectal tumours via local LRH‐1/Cyp11b1‐mediated synthesis of immunosuppressive glucocorticoids
Source: Mol Oncol. 2023 Mar 9;17(8):1545–66. doi: 10.1002/1878-0261.13414 (PMC10399709; doi:10.1002/1878-0261.13414)
Supplement: Supplementary file 1 — Fig. S1. Generation and characterization of intestine‐specific Cyp11b1‐deficient mice. Fig. S2. In vitro growth of tumour organoids. Fig. S3. Effect of dexamethasone treatment or Cyp11b1 deletion on in vivo growth of s.c. transplanted tumours. Fig. S4. Characterization of tumour immune infiltrates. Fig. S5. Effect of NR5A2 and HSD11B2 expression on colorectal patient survival and immune marker expression. [file MOL2-17-1545-s001.pdf]

# Colorectal tumors escape from immune surveillance through local synthesis of immunosuppressive glucocorticoids

## Supplementary Figures

## Suppl. Figure 1

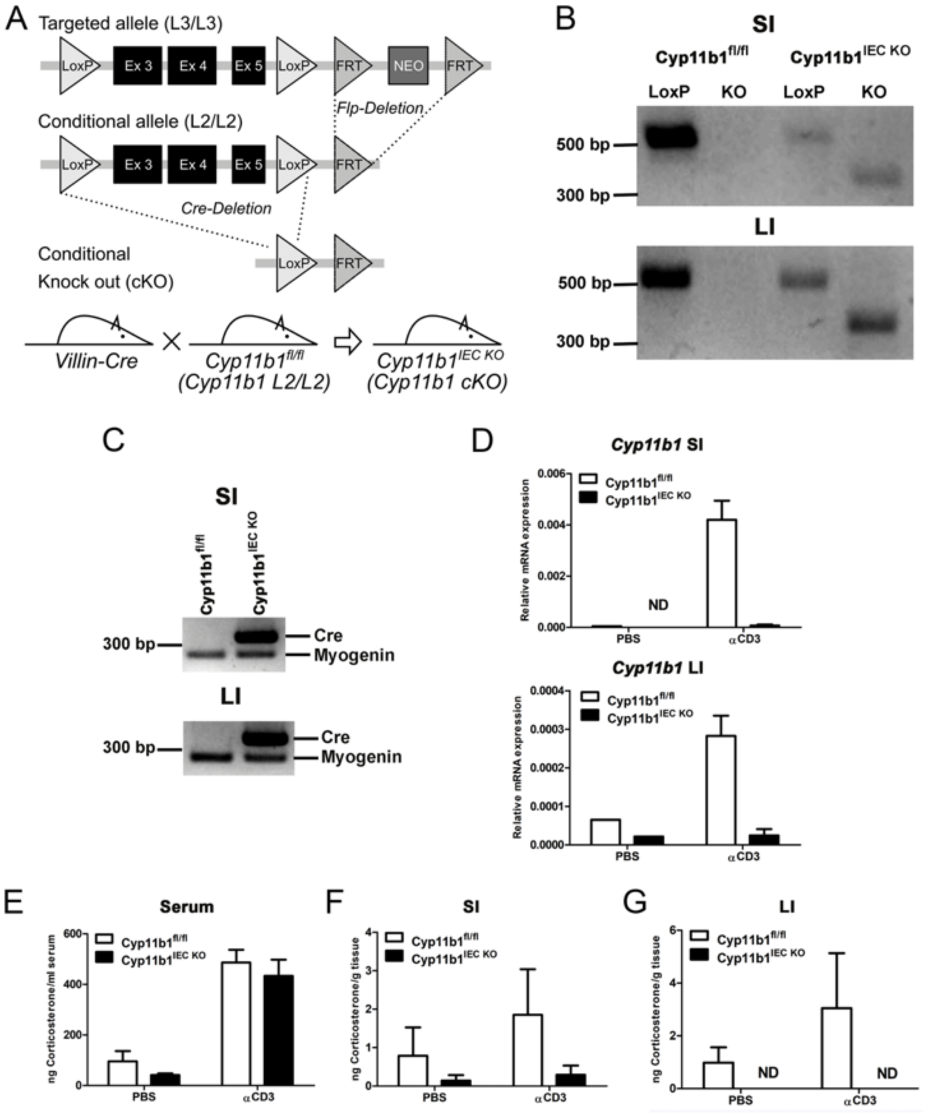


**Suppl. Figure 1: Generation and characterization of intestine-specific Cyp11b1-deficient mice.**

**A.** Targeting strategy for Cre recombinase-mediated deletion of Exon 3-5 of the Cyp11b1 gene. **B.** Detection of floxed and knockout alleles in the small (SI) and large intestine (LI) of Cyp11b1$^{fl/fl}$ and Cyp11b1$^{IEC\ KO}$ mice by PCR. **C.** Detection of Cre in small (SI) and large intestine (LI) of Cyp11b1$^{IEC\ KO}$ mice by PCR. **D.** Detection of *Cyp11b1* mRNA expression in small (SI) and large intestine (LI) of PBS or anti-CD3-injected Cyp11b1$^{fl/fl}$ and Cyp11b1$^{IEC\ KO}$ mice. **E.** Serum glucocorticoid levels of Cyp11b1$^{fl/fl}$ and Cyp11b1$^{IEC\ KO}$ mice. **F, G.** *Ex vivo* glucocorticoid synthesis in small (SI) (F) and large intestine (LI) (G) of PBS or anti-CD3 (αCD3)-injected Cyp11b1$^{fl/fl}$ and Cyp11b1$^{IEC\ KO}$ mice. d-g: n= 3 mice per group. ND, not detected.

**Suppl. Fig. 2**

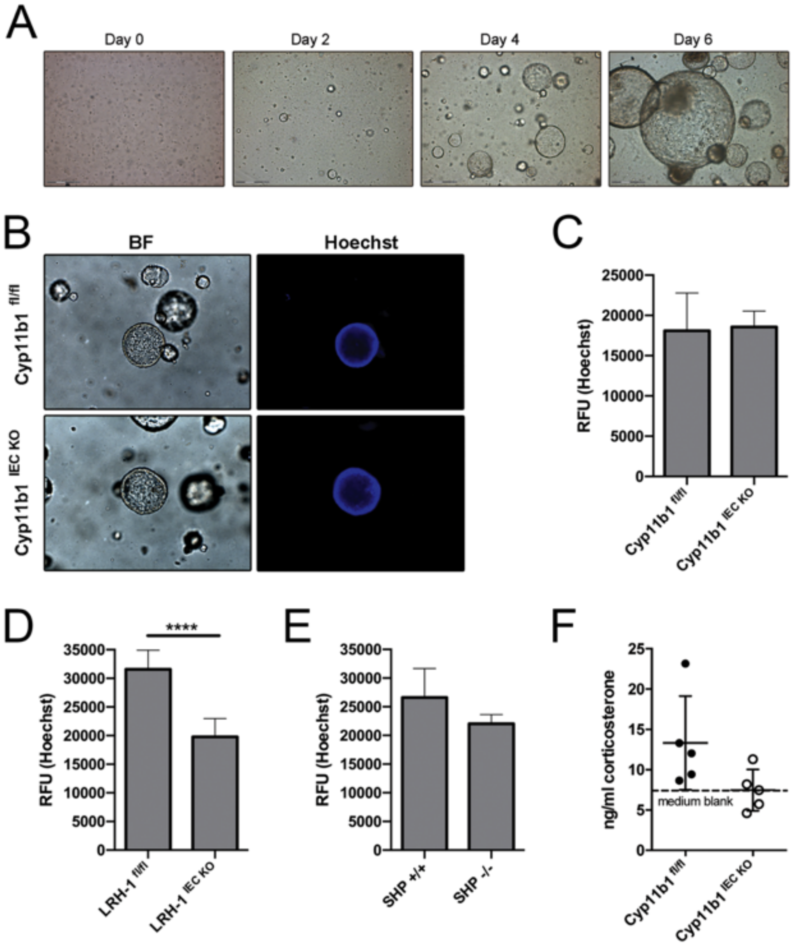


**Suppl. Figure 2:** ***In vitro* growth of tumor organoids.** Tumor cells were isolated from colonic tissue of LRH-1$^{fl/fl}$, LRH-1$^{IEC\ KO}$, SHP$^{+/+}$, SHP$^{-/-}$, Cyp11b1$^{fl/fl}$ and Cyp11b1$^{IEC\ KO}$ mice, and expanded *in vitro* as tumor organoids. **A.** Representative pictures of tumor organoid growth. **B.** Staining of tumor organoids with cell-permeable Hoechst. **C-E.** Quantification of tumor organoid growth at day 4. **C.** Cyp11b1$^{fl/fl}$ and Cyp11b1$^{IEC\ KO}$, **D.** LRH-1$^{fl/fl}$, LRH-1$^{IEC\ KO}$, **E.** SHP$^{+/+}$, SHP$^{-/-}$. Unpaired students T Test, **** $p < 0.0001$. **F.** Detection of corticosterone in the culture supernatant of Cyp11b1$^{fl/fl}$ and Cyp11b1$^{IEC\ KO}$ tumor organoids. Mean values +/- SD of n = 5 individual culture samples are shown. The dashed line indicates basal levels in culture medium alone.

## Suppl. Fig. 3

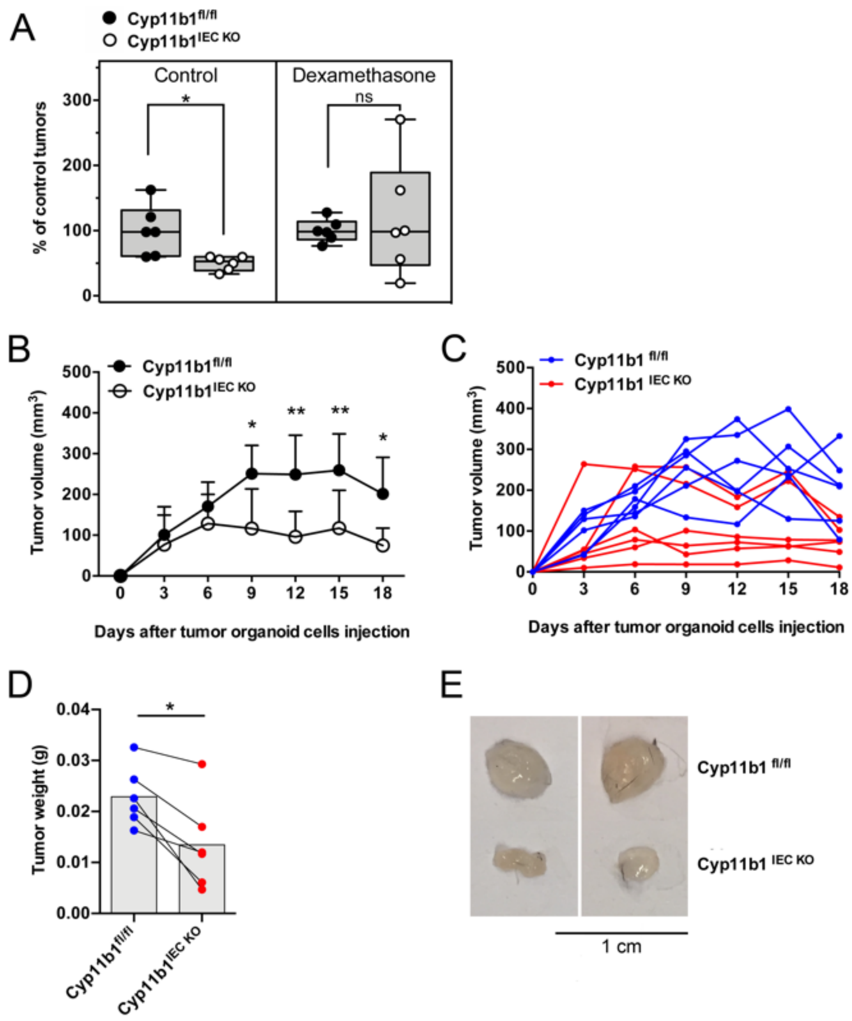


**Suppl. Figure 3: Effect of dexamethasone treatment or *Cyp11b1* deletion on *in vivo* growth of s.c. transplanted tumors.**

**A.** Tumor organoids from Cyp11b1$^{fl/fl}$ and Cyp11b1$^{IEC\ KO}$ mice were transplanted s.c. into wild type C57Bl/6 recipient mice. Recipient mice were left untreated (Control) or received 0.28 mg/ml dexamethasone in the drinking water ad libitum. At day 24, tumors were isolated and the tumor volume was analyzed (n = 6 per condition). Tumor volume was normalized to that of Cyp11b1$^{fl/fl}$ in the control group, resp. in the dexamethasone-treated group for comparison. Paired students T Test, * p < 0.05, ns not significant.

**B-E.** Cyp11b1$^{fl/fl}$ tumor organoids were transduced *in vitro* with Cre-expressing lentiviruses to delete the *Cyp11b1* gene. Resulting Cyp11b1-deficient tumor organoids (Cyp11b1 $^{IEC\text{-}KO}$) or parental tumor organoids (Cyp11b1$^{fl/fl}$) as controls were transplanted s.c. into wild type C57Bl/6 recipient mice. **B.** Tumor volume. **C.** Tumor volume of individual tumors. **D.** Tumor weight. Control tumors and Cyp11b1-deleted tumors in the same recipient mice are connected by lines. **E.** Examples of tumor pairs isolated form the same recipient mice. Paired students T Test, * p < 0.05, ** p <0.01.

## Suppl. Fig. 4

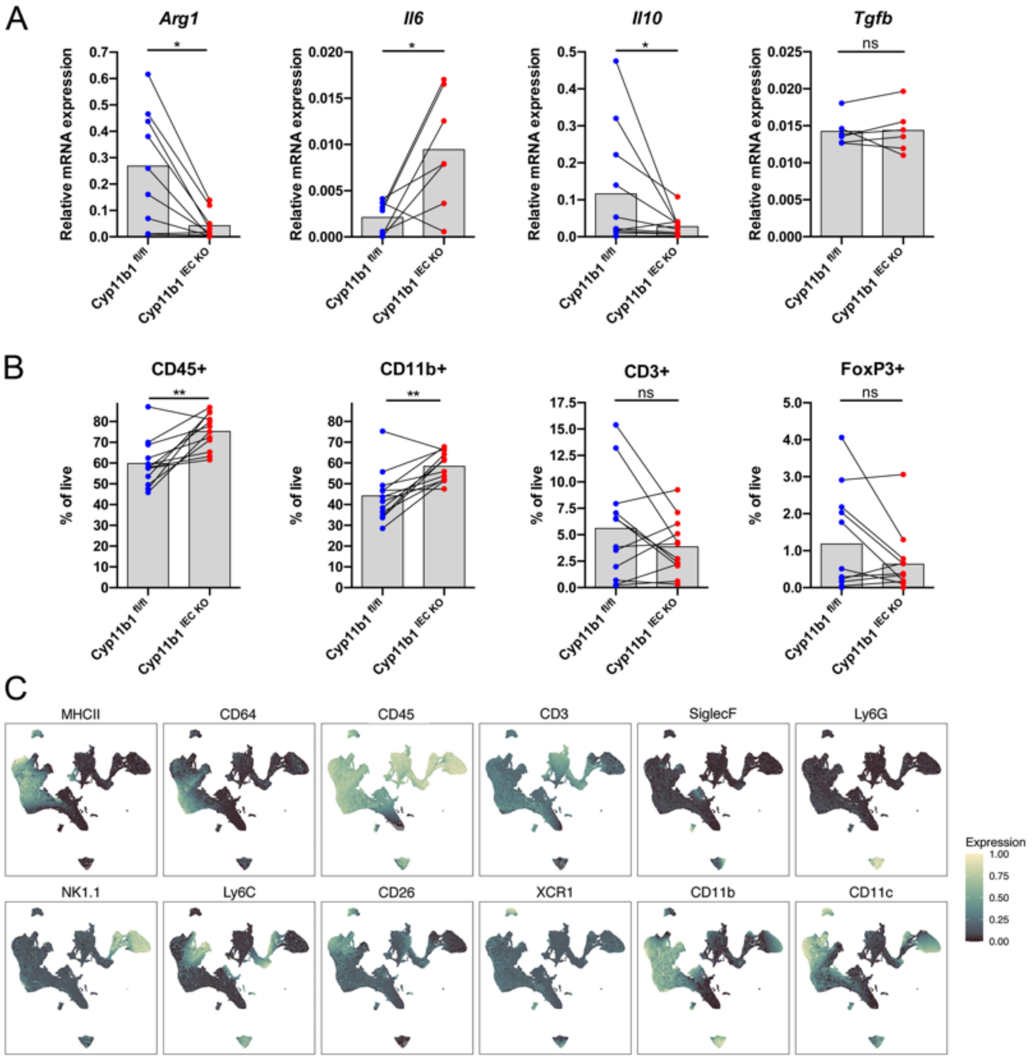


**Suppl. Figure 4: Characterization of tumor immune infiltrates.**
**A.** Detection of *Arg1*, *Il6*, *Il10* and *Tgfb* in Cyp11b1$^{fl/fl}$ and Cyp11b1$^{IEC\ KO}$ tumors transplanted into wild type recipient mice at day 12 (n = 6-11 per group). **B.** Percentage of tumor-infiltrating CD45$^{+}$, CD11b$^{+}$, CD3$^{+}$ and FoxP3$^{+}$ immune cells into Cyp11b1$^{fl/fl}$ or Cyp11b1$^{IEC\ KO}$ tumors transplanted into wild type recipient mice at day 12 (n = 12 mice per group). Data points of tumors from the same recipient mice have been connected by lines. Paired student's T test. * $p< 0.05$, ** $p< 0.01$, ns not significant. **C.** UMAP plots with 100'000 cells pooled from all tumor samples (10'000 cells randomly sampled per tumor) and overlaid heatmap lineage marker expression.

## Suppl. Fig. 5

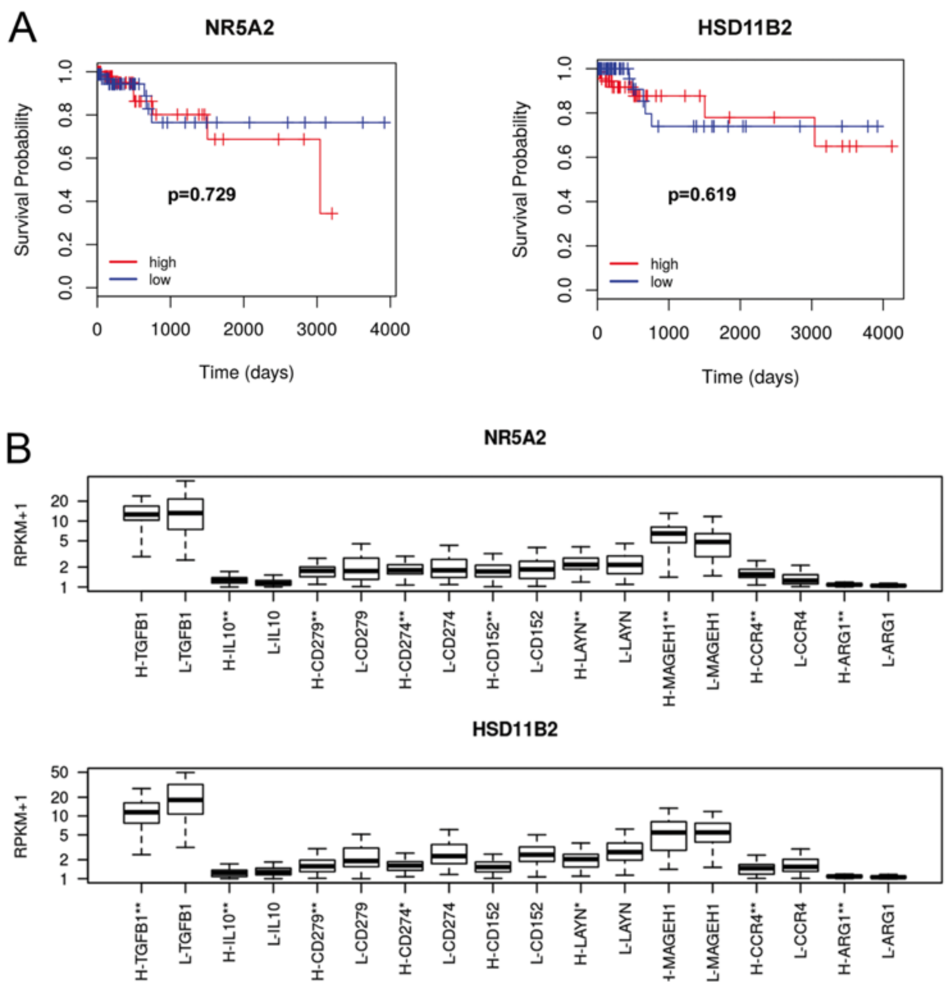


**Suppl. Figure 5: Effect of NR5A2 and HSD11B2 expression on colorectal patient survival and immune marker expression.**

**A.** Colon cancer patients were stratified into groups of the highest or lowest quartile expression of the genes *NR5A2* (LRH-1) and *HSD11B2*, and survival was analyzed. Statistical differences (p values) are indicated. **B.** Individual tumor from the highest or lowest quartile expression of *NR5A2* and *HSD11B2* were analyzed for the expression of genes related to immunoregulatory cytokines (*TGFB1*, *IL10*), immune checkpoints (*CD279* (PD1), *CD274* (PD1-L), *CD152* (CTLA4), and M2-like macrophage markers (*LAYN, CCR4, ARG1*). The data were derived from 379 colon adenocarcinoma (COAD) samples from the TCGA database using upper (high) quartile (n = 95) vs. lower (low) quartile (n = 95) expression of the given gene (H: highest quartile, L: lowest quartile of *NR5A2*, resp. *HSD11B2* expressors). The expression levels of immune genes are shown as the log RPKM - Reads per kilo base per million mapped reads. Box plots with median values, and lower and upper quartiles, and minima and maxima are shown. Wilcoxon's signed rank test. * $p < 0.05$, ** $p < 0.01$ between high and low expressors.
