# Supplementary material for: Immune escape of colorectal tumours via local LRH‐1/Cyp11b1‐mediated synthesis of immunosuppressive glucocorticoids
Source: Mol Oncol. 2023 Mar 9;17(8):1545–66. doi: 10.1002/1878-0261.13414 (PMC10399709; doi:10.1002/1878-0261.13414)
Supplement: Supplementary file 2 — Table S1. List of PCR and qRT‐PCR primers. [file MOL2-17-1545-s002.pdf]

Suppl. Table 1: List of PCR and qRT-PCR primers

Genotyping primers

Cre for	5’-GAA CCT GAT GGA CAT GTT CAG G-3’
Cre rev	5’-AGT GCG TTC GAA CGC TAG AGC CTG T-3’
myogenin for	5’-TTA CGT CCA TCG TGG ACA GC-3’
myogenin rev	5’-TGG GCT GGG TGT TAG CCT TA-3’
floxed Cyp11b1 allele for	5’-CTG AGA CAG GCA GGG ATC ATG TCT CAG-3’
floxed Cyp11b1 allele rev	5’-CCC TTG CTA TCC CAT CCA CCA AGG GG-3’
deletion product for	5’-CTG AGA CAG GCA GGG ATC ATG TCT CAG-3’
deletion product rev	5’-AGG CTG CTG CCT GTC AGG TTT TCT-3’

Primers for quantitative PCR

murine β-actin for	5’-TAT TGG CAA CGA GCG GTT CC-3’
murine β-actin rev	5’-GCA CTG TGT TGG CAT AGA GG-3’
murine cd274 for	5‘-CAG CAA CTT CAG GGG GAG AG-3‘
murine cd274 rev	5‘-TTT GCG GTA TGG GGC ATT GA-3‘
murine cd279 for	5‘-CGG TTT CAA GGC ATG GTC ATT GG-3‘
murine cd279 rev	5‘-TCA GAG TGT CGT CCT TGC TTC C-3‘
murine cd152 for	5‘-ACT GAG AGC TGT TGA CAC GG-3‘
murine cd152 rev	5‘-ACA TTC TGG CTC TGT TGG GG-3‘
murine Tnf for	5’-TAG CCC ACG TCG TAG CAA AC-3’
murine Tnf rev	5’-ACA AGG TAC AAC CCA TCG GC-3’
murine Il6 for	5’-CACAAGTCCGGAGAGGAGAC-3’
murine Il6 rev	5’-TTG CCA TTG CAC AAC TCT TT-3’
murine Il10 for	5’-GAC TTT AAG GGT TAC TTG GGT TGC-3’
murine Il10 rev	5’-GCC TGG GGC ATC ACT TCT AC-3’
murine Arg1 for	5’-GTG AAG AAC CCA CGG TCT GT-3’
murine Arg1 rev	5’-CTG GTT GTC AGG GGA GTG TT-3’

Murine Cyp11b1 and TGFβ (Tgfb) primers were obtained from Qiagen (Quantitect primers).
